# Dynamic transcriptome profiling exploring cold tolerance in forensically important blow fly, *Aldrichina grahami* (Diptera: Calliphoridae)

**DOI:** 10.1186/s12864-020-6509-0

**Published:** 2020-01-29

**Authors:** Zhuoying Liu, Han Han, Fanming Meng, Yangshuai Jiang, Jifeng Cai

**Affiliations:** 1 Department of Forensic Science, School of Basic Medical Sciences, Central South University, Changsha, Hunan China; 2 Departments of Anesthesiology and Medicine, David Geffen School of Medicine at University of California Los Angeles, Los Angeles, USA

**Keywords:** *Aldrichina grahami*, Cold tolerance, Differentially expressed genes, Transcriptome, Forensic entomology

## Abstract

**Background:**

*Aldrichina grahami* (Diptera: Calliphoridae) is a forensically important fly, which has been widely applied to practical legal investigations. Unlike other necrophagous flies, *A. grahami* exhibits cold tolerance which helps to maintain its activity during low-temperature months, when other species are usually not active. Hence, *A. grahami* is considered an important forensic insect especially in cold seasons. In this study, we aim to explore the molecular mechanisms of cold tolerance of *A. grahami* through transcriptome.

**Results:**

We collected eggs and larvae (first-instar, second-instar and third-instar) at three different temperatures (4 °C, 12 °C and 20 °C) and performed RNA-seq analyses. The differentially expressed genes (DEGs) associated with the cold-tolerance were screened out. The Venn analysis of DEGs from egg to third-instar larvae at three different temperatures showed there were 9 common genes. Candidate biological processes and genes were identified which refer to growth, and development of different temperatures, especially the chitin and cuticle metabolic process. The series-clusters showed crucial and unique trends when the temperature changed. Moreover, by comparing the results of growth and developmental transcriptomes from different temperatures, we found that DEGs belonging to the family of larval cuticle proteins (LCP), pupal cuticle protein (CUP), and heat shock proteins (HSP) have certain differences.

**Conclusions:**

This study identified functional genes and showed differences in the expression pattern of diverse temperatures. The DEGs series-clusters with increasing or decreasing trends were analyzed which may play an important role in cold-tolerance. Moreover, the findings in LCP, CUP and HSP showed more possible modulations in a cold environment. This work will provide valuable information for the future investigation of the molecular mechanism of cold tolerance in *A. grahami.*

## Background

Forensic entomology, which explores the succession pattern and developmental stages of insects found on the decomposed cadavers, has been increasingly recognized as an important tool in the medico-legal discipline [1]. The knowledge of forensic entomology offers vital clues for the postmortem interval (PMI). The developmental time of the immature stages of necrophagous flies (eggs, larvae, pupae) has been a good indicator in determining the minimum PMI (PMI_min_) [2, 3]. Comparing with other species, Calliphoridae has been investigated frequently because of predominance in colonizing corpses [4, 5]. *Aldrichina grahami*, a forensically important blow fly, is mainly distributed in Asia [6, 7]. In 1929, *A. grahami* was first reported by Aldrich in California [8]. *Aldrichina grahami* has been widely applied to practical legal investigations [9, 10] and exhibited some unique characteristics like low-temperature tolerance, which makes it active in the cold winter when other necrophagous flies almost inactive [11–13]. Several researchers are interested in development of *A. grahami* on different common temperatures, but only a few Japanese researchers explored its low-tolerance [14, 15]. In 1962, Japanese researchers found that the adults of *A. grahami* could survive when the temperature was relatively cold (2 to 7 °C) [16]. However, the hatching rates of eggs kept at this environmental condition was zero [16]. In 1985, it also had been reported in Japan that lethal time of 50% (LT_50_) of egg, larvae and pupae stages of *A. grahami* exposed to 4 °C was approximately 4, 12 and 6 days, respectively [17]. An insect species’ capacity for cold tolerance is regarded as a dominant factor for its population expansion [18] and geographical distribution [19]. It is well-known that insects always face a great challenge to survive in an extremely cold environment [20]. Recently, there are a few studies about low-temperature adaptability of other flies [21, 22], however, few researches focus on *A. grahami* and its unique characteristic at a molecular level.

Cold tolerance of insects, an intricate adaptive response which subjects to biochemical and physiological regulation, has become a hot topic in recent years [22–25]. In temperate climates, the survival of insects mainly depends on the adaptations to cold temperature [26]. For instance, the insect cuticle is secreted by epidermal cells and covers the whole surface of the body [27], which provides protection against physical injury and dehydration [28]. Previous studies have shown that the properties of the insect cuticle may be determined by the complex structural interactions between chitin polysaccharides and cuticular proteins (CPs) [29]. Moreover, extreme cold environment can affect cellular membrane integrity by inducing the transition of membrane’s phospholipid bilayer [30]. These changes in membrane fluidity alter function and activity of membrane-bound enzyme [31, 32]. Lots of attempts have been made to elucidate the mechanisms of insect cold tolerance [33, 34]. In cold winter, the metabolic activity rate of insects is generally low. And there has little changed in their state, organ development, and tissue differentiation [35]. However, their physiological metabolic processes are still active, such as energy metabolism, endocrine regulation, lipid metabolism and sugar metabolism [30, 36, 37]. In energy metabolism, it has been proved that low-molecular-weight sugars and polyols are vital intermediate metabolites and energy substances in many insect species in a cold environment [18]. Importantly, polyols and sugars generally accumulate and work as cryoprotectants at low temperature [17]. Although different insects accumulate dissimilar polyols, the increase in glycerol content is associated with the cold environment [33, 34]. The adaptability of these compounds leads to efficient resource utilization and maintain the dynamic balance of nutrition, thus providing a higher level of cold tolerance of overwintering insects [20]. In lipid metabolism, desaturation of fatty acids in membrane’s phospholipid and desaturation of triacylglycerides decrease the melting point, leading to an increasing fluidity and accessibility at low temperatures [38, 39]. Moreover, it has been reported that the inhibition of several metabolic pathways at low temperature may avoid damaging imbalance. For instance, enzyme activities and expressions in insects that regulated the physiological processes were related to survival, growth, and development [40]. Last but not least, the biochemical mechanisms of cold tolerance are reported to involve antioxidant defense [41] and aminoacyl-tRNA biosynthesis [42].

In the present study, we used RNA-seq technique to identify the cold tolerance related genes by building transcriptomes profile of eggs, and larvae (1st instar to 3rd instar) under three different temperatures (4 °C, 12 °C and 20 °C). DEGs among eggs and different instars were identified by comparative transcriptome analysis. Finally, differentially expressed LCP, CUP, and HSP in egg and larvae were examined for transcriptome data validation.

## Results

### Overview of RNA-Seq data

**Fig. 1:**
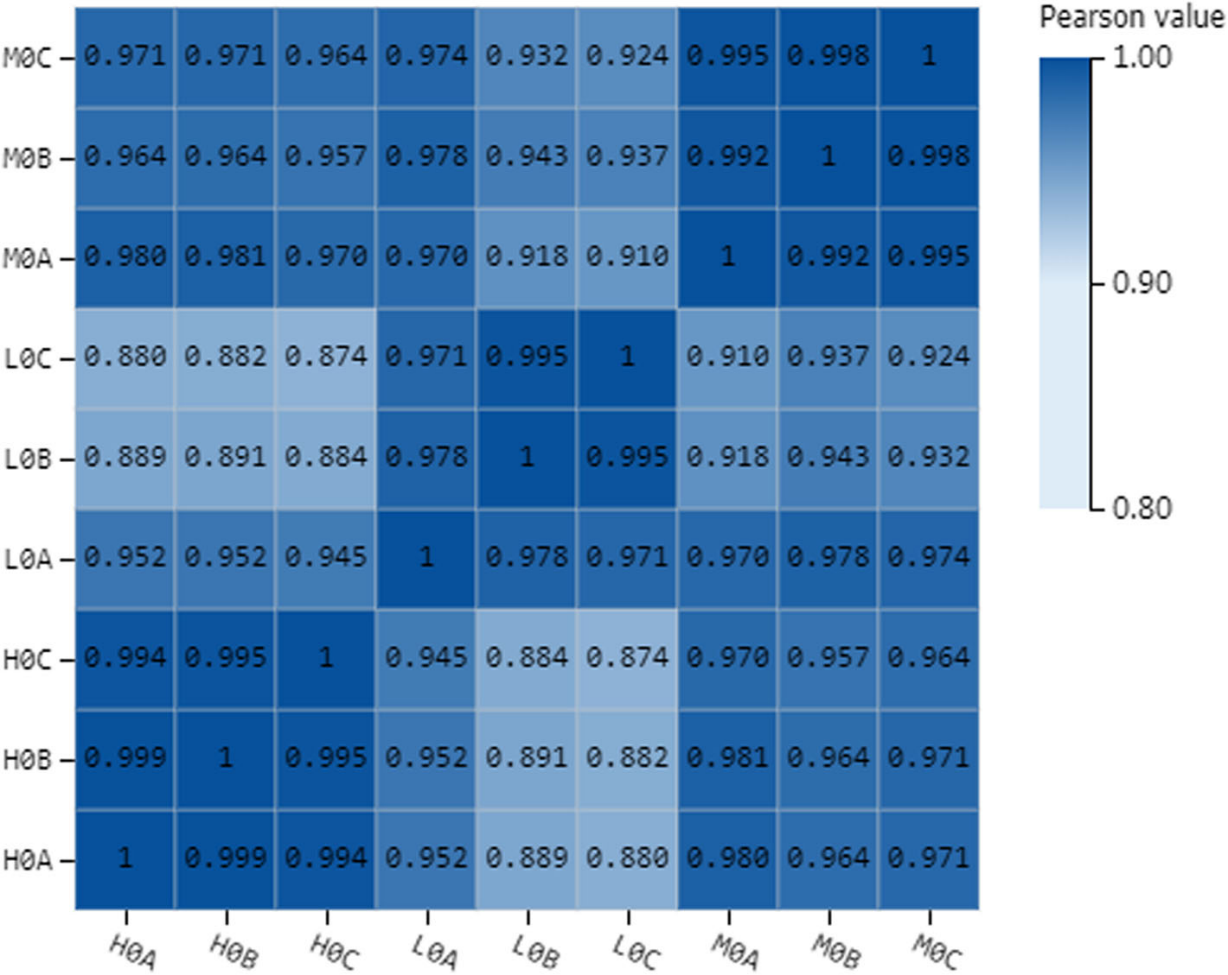
The Pearson correlation coefficient between three biological replicates of egg stage at 20 °C (H0), egg stage at 12 °C (M0), and egg stage at 4 °C (L0)

A total of 36 libraries (Additional file 1: Table S1) were sequenced from egg and larvae (first-instar, second-instar and third-instar) at three different temperatures (4 °C, 12 °C and 20 °C) of 12 groups (*n* = 3 for each), representing the egg stage at 4 °C low temperature (L0), 12 °C middle temperature (M0) and 20 °C relatively high temperature (H0); the first-instar larvae stage at low temperature (L1), middle temperature (M1) and relatively high temperature (H1); the second-instar larvae stage at low temperature (L2), middle temperature (M2) and relatively high temperature (H2); and the third-instar larvae stage at low temperature (L3), middle temperature (M3) and relatively high temperature (H3), respectively. Totally, there were 250.58 Gb clean bases was obtained. About 6.26–7.90 Gb clean bases were produced for each library. After discarding low-quality reads, RNA-seq yielded from 62.55 to 79.02 Mb clean reads with average about 90% Q30 bases for each sample, which were used for all further expression analysis. Among the total number of clean reads from 36 samples, 80.49 to 88.12% were successfully mapped against the reference *A. grahami* genome. The percentage of the unique mapping reads was 48.78 to 67.39% in each sample (Table 1). As the correlation of transcript expression level is a vital indicator for the reliability of the experimental results, we found that in egg stage, the Pearson correlation coefficient between three biological replicates of three groups in this study had high repeatability (i.e., all R^2^ ≥ 0.880; Fig. 1). Moreover, the results of larvae were showed high repeatability (Additional file 2: Figure S1).

**Table 1:** Characteristics of the reads from 36 *Aldrichina grahami* transcriptomes

Sample	Q30 value (%)	Raw reads (M)	Clean reads(M)	Total mapped reads (%)	Unique mapped reads (%)
L0A	89.45	69.69	63.91	81.29	54.73
L0B	90.22	82.12	77.16	82.58	60.65
L0C	89.20	77.40	70.83	82.12	54.15
L1A	90.12	72.69	67.06	82.13	64.65
L1B	90.28	75.19	69.32	85.17	66.64
L1C	90.55	70.18	64.73	85.70	67.39
L2A	89.95	82.39	76.31	85.21	65.99
L2B	90.16	84.58	79.02	83.33	59.70
L2C	89.19	77.54	70.93	83.33	54.57
L3A	89.84	70.51	65.84	86.10	51.28
L3B	89.06	76.88	70.39	85.39	48.78
L3C	89.08	71.94	66.17	85.19	52.53
M0A	88.78	75.20	68.53	80.54	62.78
M0B	88.66	75.20	67.48	81.05	61.90
M0C	88.53	77.71	69.78	81.62	62.64
M1A	91.40	75.19	71.82	83.85	66.46
M1B	91.08	72.69	69.13	85.27	66.74
M1C	91.22	72.69	69.27	84.80	66.42
M2A	91.42	75.19	71.65	82.99	66.29
M2B	91.50	75.20	71.98	86.06	67.29
M2C	91.18	72.69	69.08	85.61	66.89
M3A	91.06	77.70	73.75	87.21	58.27
M3B	90.99	70.18	66.55	87.30	60.65
M3C	91.07	72.69	69.20	88.12	59.31
H0A	90.53	77.70	71.96	81.26	65.66
H0B	90.72	77.70	72.06	81.02	65.23
H0C	90.66	77.70	72.13	80.49	64.98
H1A	88.56	75.20	68.01	85.90	65.15
H1B	88.97	75.20	68.47	85.68	65.33
H1C	88.91	75.20	68.27	85.20	65.00
H2A	88.36	77.71	69.97	82.92	62.59
H2B	88.34	77.71	69.85	83.22	62.47
H2C	88.79	77.71	70.03	82.84	63.20
H3A	88.62	70.19	63.20	83.75	50.76
H3B	90.66	67.68	62.55	83.60	58.01
H3C	90.57	75.19	69.33	86.37	57.51

### DEGs involved in the three different temperatures

**Fig. 2:**
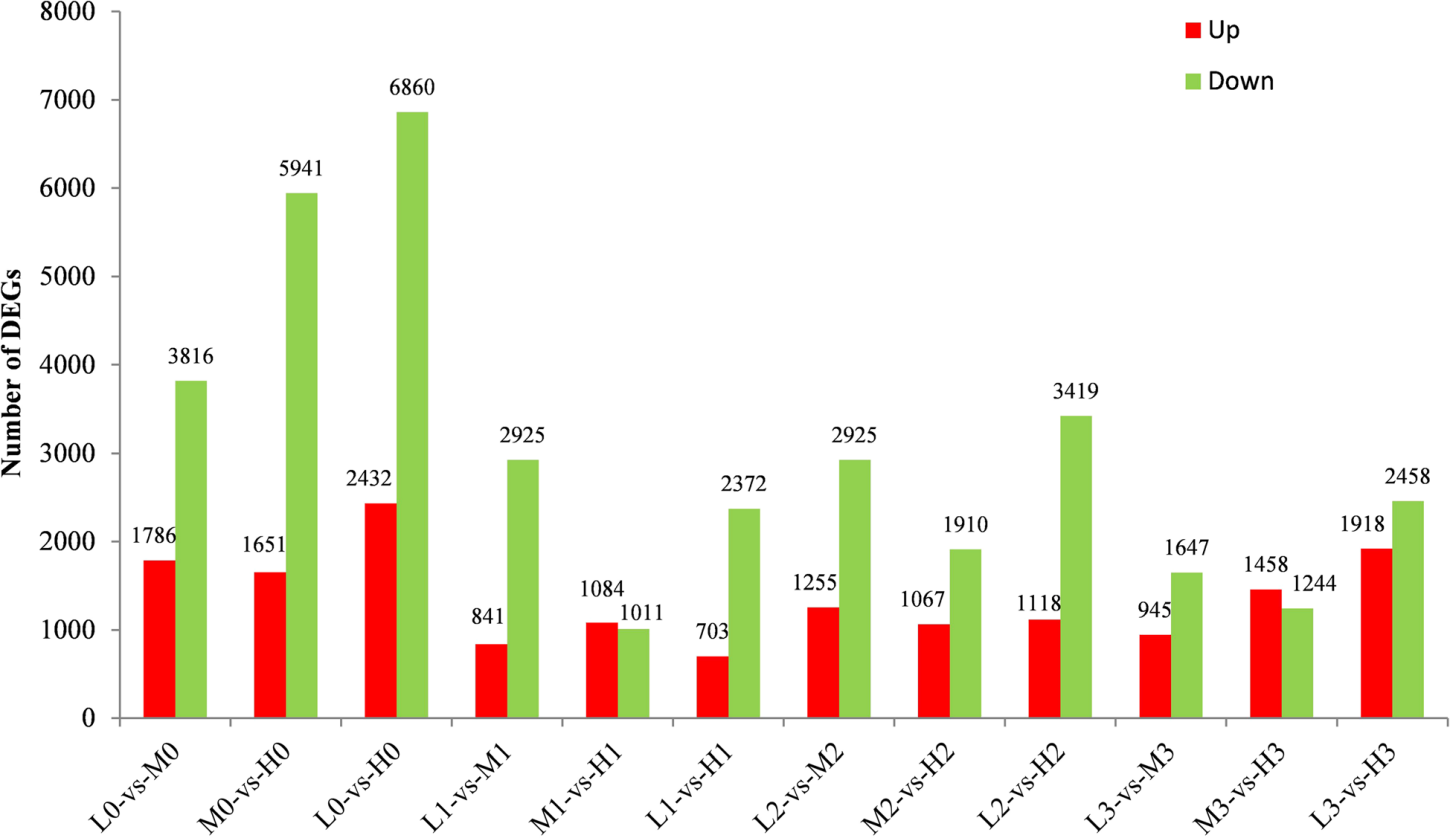
The number of differentially expressed genes between the comparison groups. Up-regulated DEGs (red), and down-regulated DEGs (green) were presented by histogram

In a set condition (*p*-value < 0.05, and fold change ≥2.0), we screened the DEGs to determine the differences under different constant temperatures. In the egg stage, there were 5602, 7592, 9292 DEGs in the comparison of L0 vs M0, M0 vs H0, L0 vs H0, respectively. In first-instar, 3766, 2095, 3075 genes were found to be DEGs in the comparison of L1 vs M1, M1 vs H1; L1 vs H1, respectively. In the second-instar and third-instar larvae stage, thousands of DEGs were also obtained in a similar comparison (Fig. 2). To sum up, the comparison of low temperature and the high temperature had the largest number of DEGs. And the egg stage was the period most affected by environmental temperature. Likewise, as the expression patterns of all the DEGs under different temperature at egg stage were analyzed (Additional file 3: Figure S2), we found out obvious differences between 4 °C and 20 °C. However, other stages didn’t show such distinct differences.

**Fig. 3:**
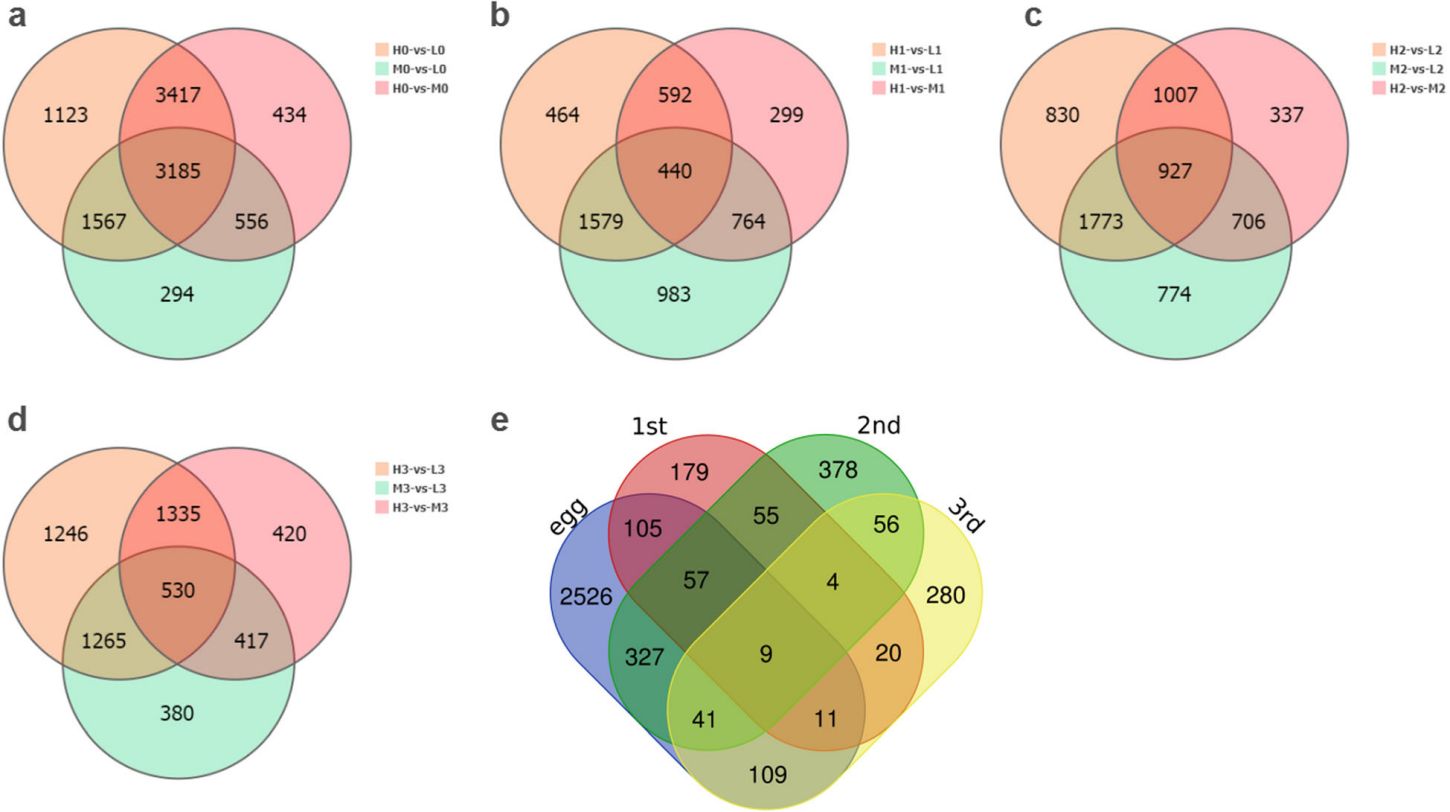
The Venn diagram showed the shared and unique genes of DEGs were statistically analyzed. **a** the Venn diagram in egg stage, **b** the Venn diagram in first-larvae stage, **c** the Venn diagram in second-larvae stage, **d** the Venn diagram in third-larvae stage, **e** the Venn diagram in (**a**, **b**, **c** and **d**)

Next, we investigated the DEGs of one development stage under three different temperatures (4 °C, 12 °C and 20 °C) at the same development stages. In egg stage, plenty of DEGs were screened in the two of the three comparisons of L0 vs M0, M0 vs H0, L0 vs H0. The number 3185 was the largest of comparisons among the four groups. In first-instar, 440 DEGs were found to be an intersection in the two of the three comparisons of L1 vs M1, M1 vs H1; L1 vs H1. In the second-instar and third-instar larvae stage, 927 and 530 DEGs were also obtained in a similar comparison (Fig. 3). Subsequently, we found out 9 DEGs (Fig. 3e) were the intersection in the two of these four comparisons, suggesting to be the most important for cold tolerance. However, none has the same trend when the temperature drops gradually in all four stages (Additional file 4: Table S2). To be mentioned, great changes had taken place in flies from eggs to mature larvae, especially in egg stage, both morphologically and genetically. Hence, DEGs of intersection in the two of these three comparisons were also worth studying.

### Series-cluster analysis and functional annotation of the clusters

**Fig. 4:**
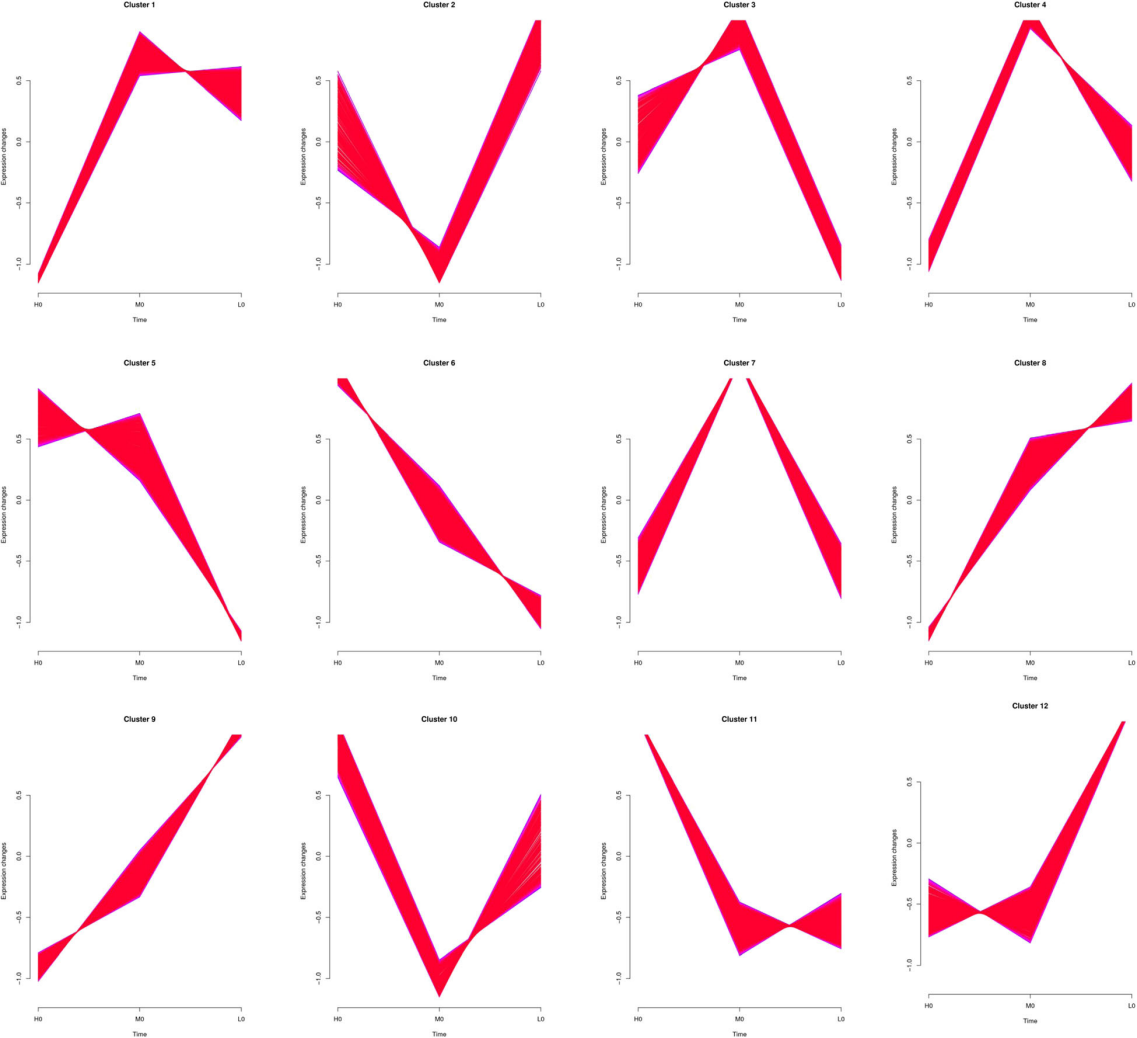
The series-clusters for DEGs in the egg stage. Each cluster of DEGs showed similar expression change in egg stage at 20 °C (H0), egg stage at 12 °C (M0), and egg stage at 4 °C (L0)

The expression patterns not only indicate the diverse and complex interactions among genes, but also suggest that genes with similar expression patterns may have similar functions when in a cold environment. In the egg libraries (0 libraries), a total of 13,356 genes were found to be DEGs. Then 12 series-clusters (Fig. 4) were obtained based on them. Each gene cluster exhibited a distinctive expression pattern. The largest group of 0 libraries is cluster 12 with 2585 (19.4%). Importantly, it can be found out that cluster 6 and cluster 9 had extremely different trends when the temperature changed. There were 1272 DEGs in the cluster and among them, 122 (9.6%) was novel genes. The cluster 6 showed directly decreasing trend when facing cold. On the contrary, cluster 9 showed a completely opposite trend. In cluster 9, there were 1615 DEGs and 626 (38.8%) was the novel one. Moreover, in the first-instar larvae libraries (1 libraries) (Additional file 5: Figure S3), the second-instar larvae libraries (2 libraries) (Additional file 6: Figure S4), and the third-instar larvae libraries (3 libraries) (Additional file 7: Figure S5), there were 12,563, 12,354, and 11,242 DEGs had been analyzed as series-cluster, respectively. There was also distinct increasing or decreasing trend in their clusters when the environment temperature was cooling down. Series-cluster analysis and functional annotation of the clusters provided crucial clues of the key DEGs that worked in cold temperature. For example, in cluster 7 of 3 libraries, we found out the third instar larval cuticle proteins (LCP) had significant changes.

### GO and KEGG pathway analysis of DEGs in 0 libraries

**Fig. 5:**
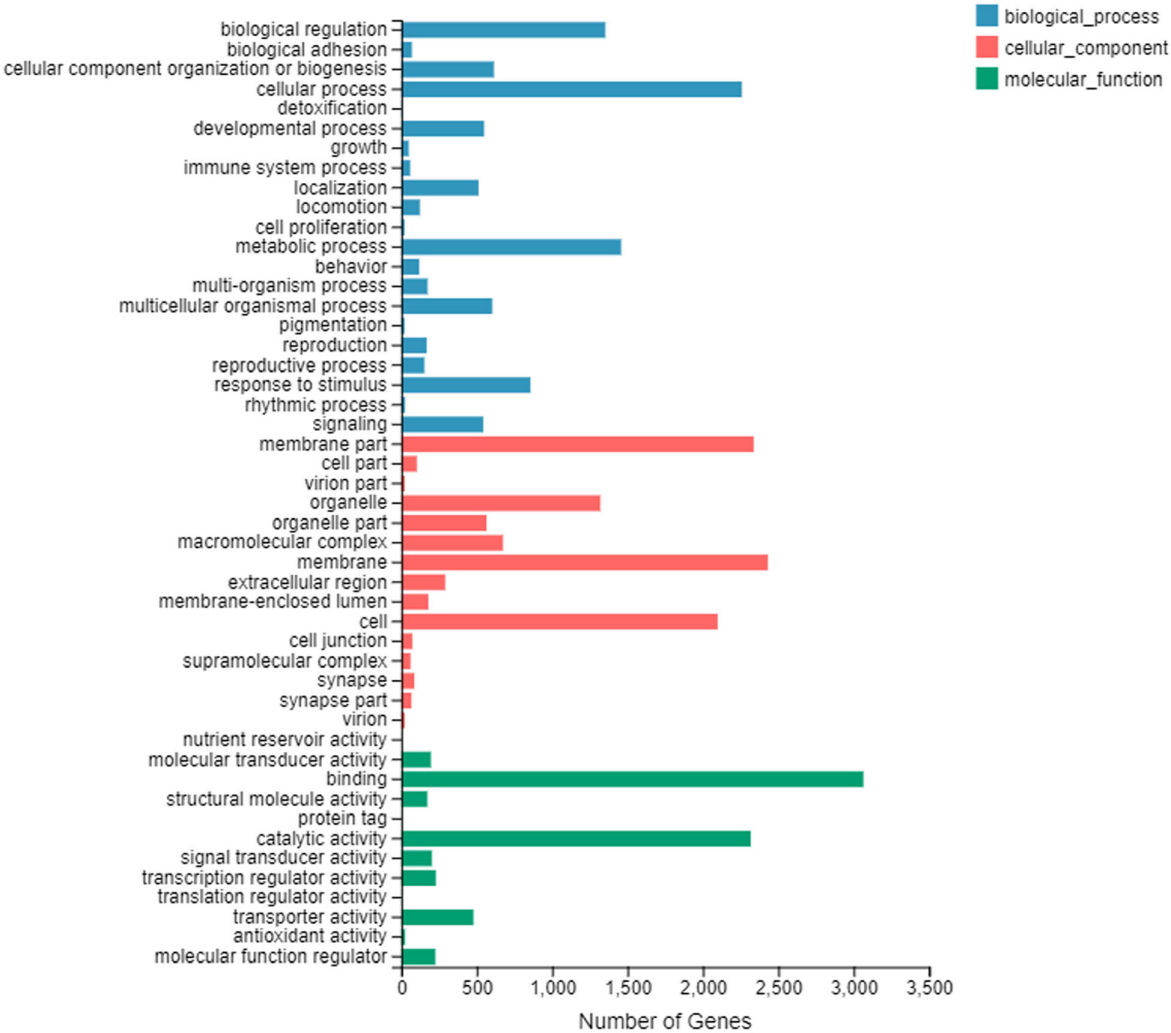
Functional annotation of assembled sequences of DEGs of egg stage at 20 °C (H0) vs egg stage at 4 °C (L0) based on gene ontology (GO) categorization. Unigenes were annotated in three categories: biological process, cellular components, and molecular functions

A total of 7250 DEGs were annotated into GO terms involved in the egg stage. Undoubtedly, the L0 vs H0 has the largest number of DEGs. Among them, 1992 (31.4%) novel genes were found out. DEGs were classified into 48 subcategories within three standard categories (molecular functions, biological processes and cellular components) (Fig. 5). “Cellular process” and “metabolic process” were the most enriched in the biological process domain. In cellular component category, “membrane” and “membrane part” were the highest enriched, while “binding” and “catalytic activity” were the most enriched in the molecular function category. Meanwhile, the significant enriched GO terms (*p*-value < 0.05) involved in the egg stage were determined from the DEGs of L0 vs M0, M0 vs H0, L0 vs H0 (See for a full list of GO terms in Table 2). DEGs of chitin metabolic process, glucosamine-containing compound metabolic process, amino sugar metabolic process and structural constituent of cuticle were all significantly enriched in L0 vs M0, M0 vs H0, L0 vs H0.

**Table 2:** GO functional enrichment analysis related with temperature of the DEGs of H0 vs L0, H0 vs M0 and M0 vs L0

Tissue comparison	GO Term	Rich Ratio	*P*-value
H0vsL0	chitin metabolic process	0.78	4.14E-09
	glucosamine-containing compound metabolic process	0.78	5.92E-09
	amino sugar metabolic process	0.77	1.20E-08
	chitin binding	0.78	1.41E-08
	structural constituent of cuticle	0.76	1.39E-07
	response to stimulus	0.60	2.01E-06
	G-protein coupled receptor activity	0.73	0.0001
	DNA-dependent DNA replication	0.80	0.0006
	circadian rhythm	0.83	0.0016
	chitin-based cuticle sclerotization	0.86	0.0030
	RNA metabolic process	0.63	0.0330
H0vsM0	glucosamine-containing compound metabolic process	0.73	8.75E-12
	aminoglycan metabolic process	0.70	1.71E-11
	amino sugar metabolic process	0.72	1.76E-11
	chitin metabolic process	0.72	2.33E-11
	chitin binding	0.70	1.60E-09
	structural constituent of cuticle	0.63	1.76E-05
	anatomical structure development	0.50	0.0012
	muscle cell development	0.80	0.0017
	DNA binding	0.51	0.0036
	chitin-based cuticle sclerotization	0.76	0.0041
	cuticle development	0.62	0.0057
M0vsL0	structural constituent of cuticle	0.76	2.15E-27
	glucosamine-containing compound metabolic process	0.68	2.68E-21
	amino sugar metabolic process	0.68	5.85E-21
	chitin metabolic process	0.68	1.42E-20
	aminoglycan metabolic process	0.64	4.45E-20
	chitin binding	0.64	1.09E-15
	G-protein coupled receptor activity	0.54	2.02E-06
	muscle cell development	0.70	0.0003
	cuticle development	0.52	0.0003
	circadian rhythm	0.62	0.0004
	chitin-based cuticle sclerotization	0.67	0.0007

**Fig. 6:**
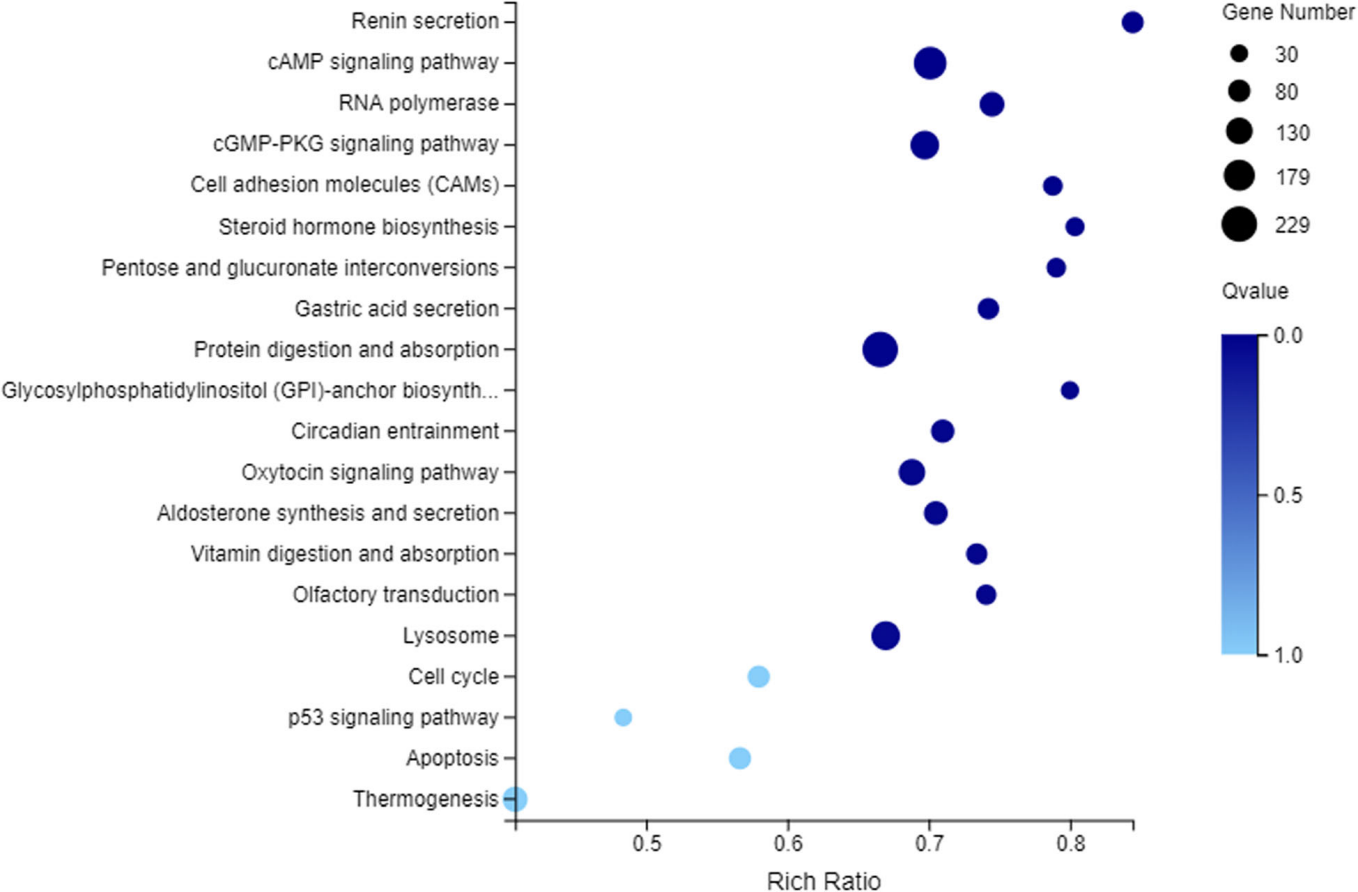
KEGG significant enrichment analysis for DEGs between egg stage at 20 °C (H0) and egg stage at 4 °C (L0) of *A. grahami*

Meanwhile, KEGG pathway related to temperature changes were also recognized (Fig. 6), including cAMP signaling pathway, cGMP-PKG signaling pathway, pentose and glucuronate interconversions, oxytocin signaling pathway and circadian entrainment, etc. Importantly, the cAMP and cGMP-PKG signaling pathway, which are involved in energy metabolism and cell growth and differentiation, are suggested to be important during the temperature changes in *A. grahami.* To be mentioned, endocrine system and digestive system might play a vital role in a cold environment.

### GO and KEGG pathway analysis of DEGs in 1 and 2 library

A total of 5121 DEGs and 6354 DEGs were annotated into GO terms involved in the first-instar and the second-instar, respectively. DEGs of L1 vs H1 (Additional file 8: Figure S6) were classified into subcategories within three standard categories. “Cellular process” and “biological regulation” were the most enriched terms in the biological process domain. Similarly, in cellular component category, “membrane” and “membrane part” were the highest enriched, while “binding” and “catalytic activity” were mostly enriched terms in the molecular function category. Moreover, the significantly enriched GO terms (*p*-value < 0.05) involved in the first-instar larvae stage were determined from the DEGs of L1 vs M1, M1 vs H1; L1 vs H1 (Additional file 9: Table S3). Response to stimulus, circadian rhythm, regulation of glucose metabolic process, chitin metabolic process, glucosamine-containing compound metabolic process, structural constituent of cuticle and amino sugar metabolic process were all significantly enriched in the DEGs of L1 vs M1, M1 vs H1; L1 vs H1.

KEGG pathway related to temperature changes was also recognized (Additional file 10: Figure S7), including protein digestion and absorption, endocrine resistance, cGMP-PKG signaling pathway, cAMP signaling pathway, longevity regulating pathway and circadian entrainment, etc. In addition, the results of GO (Additional file 11: Figure S8 and Additional file 12: Table S4) and KEGG pathway (Additional file 13: Figure S9) analysis in 2 library were similar to that in 1 library.

### GO and KEGG pathway analysis of DEGs in 3 libraries

A total of 5593 DEGs were annotated into GO terms involved in the third-instar. DEGs of L3 vs H3 were classified into subcategories within three standard categories (Additional file 14: Figure S10). “Cellular process” and “biological regulation” were the most enriched in the biological process domain. “Membrane” and “membrane part” were the highest enriched in cellular component category. And “binding” and “catalytic activity” were the most enriched in the molecular function category. Moreover, the significant enriched GO terms (*p*-value < 0.05) involved in the third-instar larvae stage were determined from the DEGs of L3 vs M3, M3 vs H3 and L3 vs H3 (Additional file 15: Table S5). Unlike 0, 1 and 2 libraries, DEGs of chitin metabolic process, chitin binding, structural constituent of cuticle were not obviously enriched in 3 libraries. DEGs of response to starvation, energy reserve metabolic process, response to stimulus, glucosamine-containing compound metabolic process and amino sugar metabolic process were all significantly enriched in 3 libraries.

Furthermore, KEGG pathway related to temperature changes were also recognized (Additional file 16: Figure S11), including protein digestion and absorption, glycosphingolipid metabolism, PPAR signaling pathway, PI3K-Akt signaling pathway and RNA polymerase, etc.

### Gene-co-expression

**Fig. 7:**
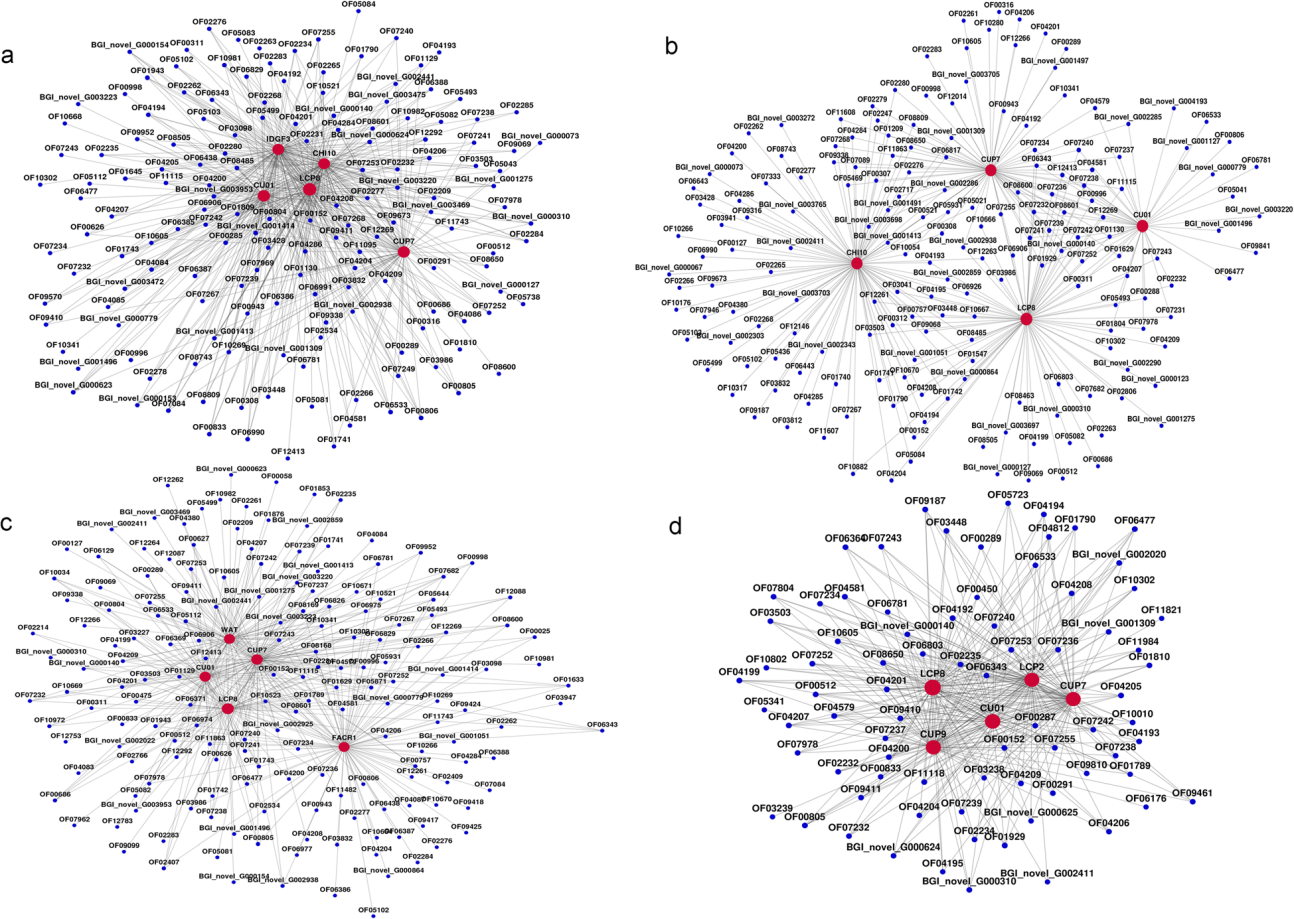
The gene co-expression net-work of DEGs in the cold temperatures (4 °C) was analyzed. **a** the gene co-expression net-work in egg stage at 4 °C (L0), **b** the gene co-expression net-work in first-instar larvae stage at 4 °C (L1), **c** the gene co-expression net-work in second-instar larvae stage at 4 °C (L2), **d** the gene co-expression net-work in third-instar larvae stage at 4 °C (L3)

The gene co-expression net-work of DEGs in the cold temperatures was analyzed (Fig. 7). There are several core genes with the highest degrees connect with most adjacent genes in the network, which are frequently identified as key indicators. Of these, DEGs which were involved in chitin metabolic process (LCP2, LCP8 and CHI10) and structural constituent of cuticle (CU01, CUP7 and CUP9) showed the strongest relationship in net-work. Next, gene co-expression net-work analysis of DEGs in 12 °C (Additional file 17: Figure S12) and 20 °C (Additional file 18: Figure S13) were also constructed. From the results of the high temperature group, there were different core genes compared with low temperature group, but still involved in chitin metabolic process, lipid metabolism, etc. Gene co-expression analysis could provide the vital genes that may regulate the adaptations in response to a cold environment.

### Validation of sequencing data

**Fig. 8:**
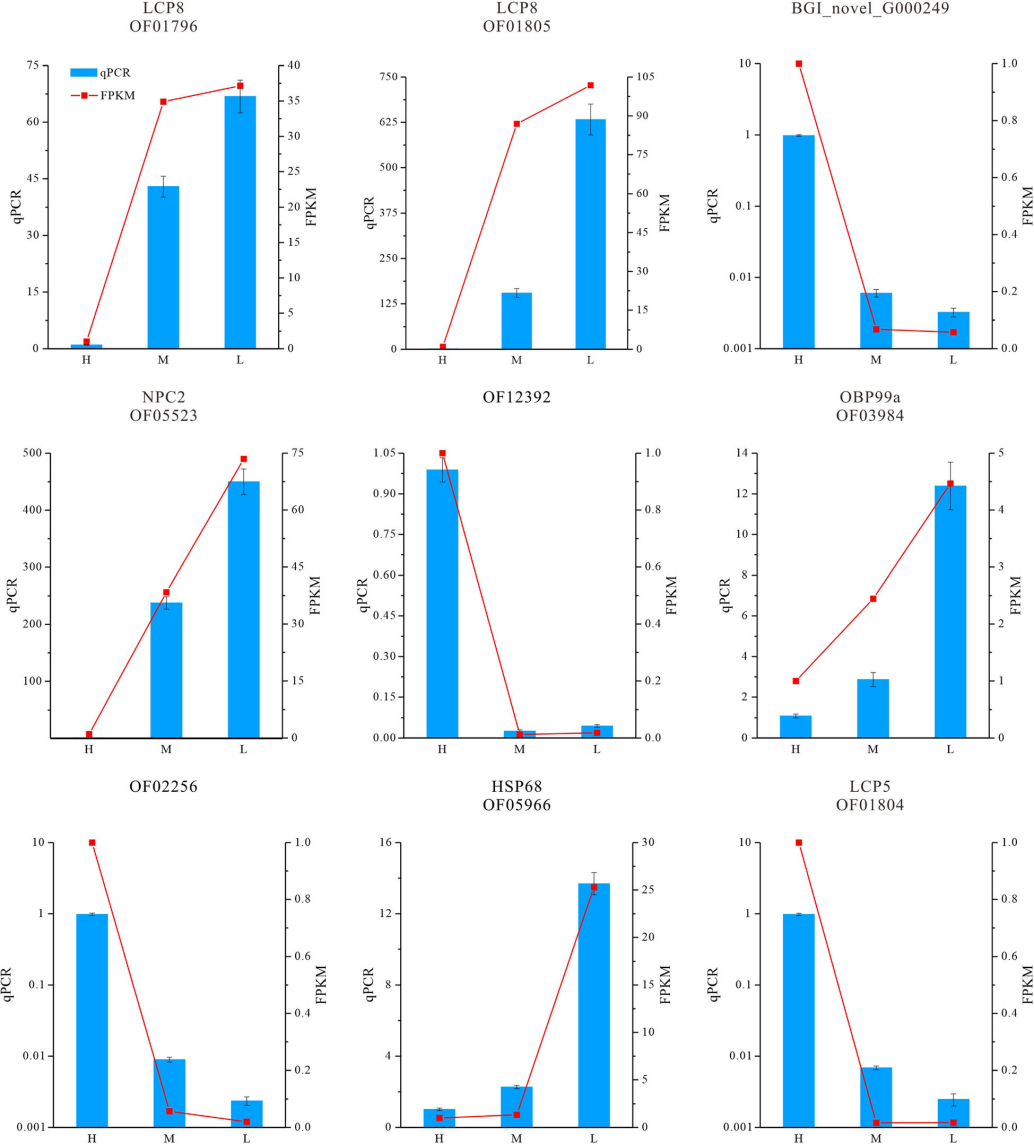
RNA-seq data validation by quantitative real-time PCR (qRT-PCR). The histograms show 9 DEGs of *A. grahami*, the red line charts show the FPKM values of these unigenes, and blue bars show the qPCR results, represent the mean ± SD of three biological replicates. The left Y-axis indicates the relative expression levels calculated by qPCR and the right Y-axis indicates the FPKM values of RNA-seq data

A total of 9 DEGs were selected for the purpose of validating whether the sequencing and analysis were reliable. Four DEGs were selected for qRT-PCR quantification from RNA sample in 0 library. Similarly, five DEGs were selected from RNA sample in 3 library. The details of those unigenes and primer pairs used in this study are shown in Additional file 19: Table S6. A comparative analysis of all the selected genes showed a similar expression pattern in the qRT-PCR analysis as observed in RNAseq data (Fig. 8). To be mentioned, the expression trends of several crucial DEGs, such as LCP8, LCP5, HSP68 and OBP99a, were as expected.

## Discussion

### Developmental transcriptomes comparisons between 4 °C and 20 °C

It is known that 20 °C is the optimum growth temperature for the development of *A. grahami*, while 4 °C is an extreme low temperature [16, 17]. Hence, the developmental transcriptomes profile of separated instars under two different temperatures were compared to explore genes that related to the cold tolerance. Many biological processes were enriched in both 4 °C and 20 °C libraries. DEGs of Chitin metabolic process, structural constituent of cuticle, glucosamine-containing compound metabolic process, and amino sugar metabolic process were all significantly enriched in H0 vs L0, H1 vs L1, and H2 vs L2. Chitin and sugar metabolic process showed an important role in a cold environment. In the results of gene-co-expression, it also showed the importance of Chitin metabolic process. It was consistent with that chitin is a component of insect cuticle [29], which could protect the body of insect [27], and CPs and sugar were vital intermediate metabolites in many insect species in a cold environment [10]. Moreover, our results also suggested that endocrine system and digestive system played vital roles in cold tolerance. Especially, the cAMP and cGMP-PKG signaling pathway were all significantly enriched in the DEGs of H0 vs L0, H1 vs L1, and H2 vs L2, indicating the involvement of energy metabolism [43, 44] and cell growth and differentiation [45, 46]. However, unlike 0, 1 and 2 libraries, chitin metabolic process, chitin binding, structural constituent of cuticle were not obviously enriched in 3 libraries. In the third-instar larvae, response to starvation, energy reserve metabolic process, glucosamine-containing compound metabolic process and amino sugar metabolic process were all significantly enriched. This may be caused by energy saving for the pupal stage [47].

### DEGs may play crucial roles in cold tolerance in *A. grahami*

Firstly, we found that 9 DEGs were the intersection-based common genes in all libraries, suggesting their involvement in cold tolerance. Unfortunately, none showed consistent trend in the four developmental stages in our studies when different temperatures. This may be caused by the morphological and genetic changes taken place from eggs to mature larvae in flies [48–50]. In addition, we explored the expression patterns in each library via a cluster way, in which the same cluster not only indicates the diverse and complex interactions among genes, but also suggests that genes may have similar functions when in a cold environment. Twelve series-clusters were obtained in each library. We found that cluster 6, cluster 11, cluster 11, cluster 2 had directly decreasing trend when facing cold in 0 library, 1 library, 2 library, 3 library, respectively. On the contrary, cluster 9, cluster 8, cluster 4, cluster 11 had a continuous increasing trend in 0 library, 1 library, 2 library, 3 library, respectively. As we aimed to explore the cold tolerance, it was an effective way to search for the key DGEs in these clusters. Moreover, DEGs which were involved in chitin metabolic process (LCP2, LCP8 and CHI10) and structural constituent of cuticle (CU01, CUP7 and CUP9) showed the strongest relationship in net-work, indicating that cuticle protein family may play significant roles in cold tolerance. It is noticeable that lipid metabolism (WAT and FACR1) also played an important role when facing cold. Therefore, we proposed that LCP, CUP and HSP might be responsible for cold-tolerance in *A. grahami*.

Although chitin is one of the vital component of the cuticle, there are various larval cuticle proteins, providing protection in cold [51, 52]. It is reported that when exposed to cold or some other environmental stresses, insects can synthesize heat shock proteins, which function as molecular chaperones in protecting cellular proteins [53]. Moreover, HSPs have also been reported in *Drosophila* [54, 55], *Bemisia tabaci* [53], *Bombyx mori* [56], etc. Surprisingly, we found that odorant-binding proteins (OBPs) had significant increases in H3 vs L3, but no differences in H0 vs L0. OBPs are the key step in the insect olfaction [57], suggesting that *A. grahami* need better olfactory sensation to search for food in cold weather. Finally, we chose 9 unigenes to examine our hypothesis via qPCR method, which were involved in synthesis of cuticle proteins, heat shock proteins, and odorant-binding proteins. These results showed the same trend with transcriptome data (Fig. 8).

The developmental stages of insect include eggs, larvae, pupae and adult. In this study, we focused on eggs and larvae stages depending on the following reasons: Firstly, low-temperature tolerance at egg stage directly affects survival rate. Larvae are also susceptible to cold temperature, while pupae and adult have their outermost shell or fluff to keep warm. Secondly, gene changes in eggs and larvae stages are mostly continuous. Dramatic changes would take place in flies from larvae to adult, both morphologically and genetically. Moreover, as the individual differences of transcriptome in adults are various, it is insensible to explore its features by adults at first. Hence, we chose eggs and larvae stages to explore the cold tolerance in *A. grahami.* Apart from the feature exploration, we also focused on the developmental expression profiles subsequently. Then we selected the key DEGs related to development time, which could be applied in PMI_min_ deduction in the future.

Importantly, when study the low temperature on the development of the blow fly, apart from low temperature, several factors should be taken into consideration. Firstly, the effects of constant temperature and fluctuating temperature on the development of blow fly [15]. Secondly, the insects from different geographic regions might have different cold tolerance [12]. Lastly, as blow fly is a kind of heterothermy animal, its larvae often gathering and feeding together and can produce heat [58, 59]. The larval mass effect can help the larvae resist the cold environment. Hence, it is sensible to consider and avoid these aspects when exploring relative studies. To be mentioned, *A.grahami* has been widely applied to practical forensic investigations [9, 10]. And the low-temperature tolerance of *A.grahami* [16, 17] makes it more important among necrophagous flies, especially in the cold environment. However, it cannot be ignored that 4 °C is an extreme low temperature. Under this condition, the egg of *A.grahami* indeed requires a long period to hatch. Moreover, it is also difficult for the adult to emerge from pupae. Hence, it would be meaningful to explore a low temperature between 4 °C and 12 °C in the future, at which the *A. grahami* could fly, mate, oviposit and develop slowly but more naturally.

## Conclusion

In this study, we provide a new insight into transcriptional profiles of egg and larvae of *A.grahami* under different temperatures condition. Various pathways and biological processes were annotated that related with dynamic gene expression of *A. grahami* under low temperature treatment, like chitin metabolic process, structural constituent of cuticle, glucosamine-containing compound metabolic process, and amino sugar metabolic process, etc. Differentially expressed gene cluster and molecular network also indicated a complicate mechanism beneath the cold-tolerance of *A.grahami*. Furthermore, the DEGs, such as LCP, CUP and HSP, showed more possible modulations in a cold environment. The accumulated knowledge about *A. grahami* biology makes it to be a promising model for detailed mechanism researches on those forensically important fly species. This work provided valuable information for future research on the mechanism of cold-tolerance of *A. grahami.*

## Methods

### Specimen collection and fly rearing

The oriental adult specimens of *A. grahami* were obtained using pork in Changsha city (28°12′N, 112°58′E), Hunan province, China, in March 2016. The adult flies were maintained in a rearing box (35 × 35 × 35 cm^3^) at 25 °C with 70% humidity and 12: 12 h light: dark photoperiod. One dish contained 1:1 mixture of sugar and milk powder as food for adults, and another dish contained 15 g of fresh pig lung to induce egg laying. After fresh pig lung was set up, the egg laying was checked and eggs were collected every half hour. Each egg was transferred to a new dish in an artificial climate box until its wandering stage. Three artificial climate boxes were used in our study at constant temperatures of 4 °C, 12 °C, and 20 °C, respectively. Due to the timeframe for development of eggs varies greatly at different temperatures, we have examined the time for egg development under each temperature in our preliminary study. We found that it took approximately 10 days, 52 h, and 28 h to develop into first-instar larvae (from eggs) at 4 °C, 12 °C, and 20 °C, respectively. Therefore, we used the following time points to represent egg stage as 5 days for 4 °C (L0), 26 h for 12 °C (M0), and 14 h for 20 °C (H0). Samples were harvested at designed time points and snap frozen in liquid nitrogen and then stored at − 80 °C. Three biological replicates of each group were used for RNA extraction and transcriptome sequencing.

### RNA extraction and library preparation for transcriptome sequencing

The Trizol method (Invitrogen, USA) was adopted to extract the total RNA of samples collected. The RNA quality was assessed by formaldehyde agarose gel electrophoresis and was quantitated spectrophotometrically (NanoDrop 2000). DNase I (TakaRa, Japan) was used for RNA purification. Double-stranded cDNA was synthesized using random hexamer-primers, reverse transcriptase, DNA polymerase I and RNaseH by taking short fragments as templates. Next, double-stranded cDNA was subsequently subjected to end-repair and ligation with adapters. These modified products were enriched with PCR to construct the final cDNA library. After test the quality of libraries, they were loaded onto the flow cell channels of the BGISEQ500 platform (BGI-Shenzhen, China).

### Sequence reads mapping and assembly

Firstly, raw reads of fastq format were filtered by removing reads containing adaptors, reads containing poly-N and low quality reads, then high-quality clean reads were obtained (reads contain 20% base quality lower than Q20). At the same time, Q20, Q30, GC-content and sequence duplication level of the clean data were calculated. All the succeeding analyses were carried out using high quality clean reads. Clean reads were mapped to the *Aldrichina grahami* genome assembly (NCBI: PRJNA513084) by using HISAT2 [60] with following parameter: --dta --phred64 unstranded --new-summary -x index − 1 read_r1–2 read_r2 (PE).

### Differential expression analysis

Differential expression analysis of two groups was performed using the parameter FPKM (Fragments per kilobase of transcript per million mapped reads), which was applied to quantify the gene expression levels. HTseq [61] was used for count calculation and FPKM was calculated with NCBI gtf file through gene length annotation. The DEGseq package was applied to filter the DEGs with a fold change > 2 or fold change < 0.5, and false discovery rate (FDR) < 0.05 [62].

### GO enrichment and KEGG pathway enrichmenanalysis

Gene ontology (GO) analysis was performed to facilitate elucidating the biological implications of unique genes in the significant or representative profiles, which is conducive to find those GOs with more concrete function description in our study [63]. KEGG [64] is a database resource for understanding high-level functions and utilities of the biological system. Pathway analysis was used to find out the significant pathways of the DEGs via the KEGG database. A Fisher exact test was used to find the vital enrichment pathway with the threshod of the significance of *p*-value < 0.05 and FDR < 0.05, which could find those crucial pathways in our study.

### Classification and co-expression of DEGs

Series cluster analysis was performed using STEM [65] to classify the DEGs in twelve clusters based on the FPKM change tendency of genes on three different temperatures of each developmental stage. Fisher’s exact test and the multiple comparison tests were used to calculate the significant levels of profiles [66, 67]. Gene co-expression network analysis was performed to track the interactions among the DEGs, according to the dynamic expression changes on three different temperatures of each developmental stage. Pearson correlation was applied to each pair of genes and the significantly correlated pairs were used to construct the network [68].

### Quantitative real-time PCR analysis

To assess the reliability of the sequencing and analysis by quantitative real-time PCR (RT-qPCR), we used the same RNA samples for transcriptome sequencing. The RT-qPCR reactions were carried out on a 7500 Real-Time PCR System (Applied Biosystems) using the 2 × T5 Fast qPCR Mix (SYBR Green I) (Qingke Biotechnology Co., Ltd. Hunan, China) according to the manufacturer’s instructions. Every RT-qPCR amplification mixtures (20 μL) contained 2 × T5 Fast qPCR Mix (10 μL), 50 × ROX Reference Dye II (0.4 μL), each forward and reverse primers (0.8 μL), diluted cDNA (2 μl) and RNase-free water (6 μL). The PCR reaction was set as follows: an initial denaturation at 95 °C for 1 min, followed by 40 cycles of 95 °C for 10 s; 60 °C for 10 s; 72 °C for 15 s. Relative gene expression levels were calculated by 2^-△△Ct^, GST1 were selected as internal reference genes [69]. The primers of all selected genes were designed by Primer Premier 5.

## Supplementary information

**Additional file 1: Table S1**. Specimen collection of *Aldrichina grahami*.

**Additional file 2: Figure S1**. The Pearson correlation coefficient between three biological replicates. (a) the Pearson correlation coefficient between three biological replicates of first-instar larvae at 20 °C (H1), first-instar larvae at 12 °C (M1), and first-instar larvae at 4 °C (L1), (b) the Pearson correlation coefficient between three biological replicates of second-instar larvae at 20 °C (H2), second-instar larvae at 12 °C (M2), and second-instar larvae at 4 °C (L2), (c) the Pearson correlation coefficient between three biological replicates of third-instar larvae at 20 °C (H3), third-instar larvae at 12 °C (M3), and third-instar larvae at 4 °C (L3).

**Additional file 3: Figure S2**. The expression patterns of all the DEGs under egg stage at 20 °C (H0), egg stage at 12 °C (M0), and egg stage at 4 °C (L0).

**Additional file 4: Table S2.** Expression level of the 9 DEGs in *Aldrichina grahami*.

**Additional file 5: Figure S3**. The series-clusters for DEGs in the first-instar larvae stage. Each cluster of DEGs showed similar expression change in first-instar larvae at 20 °C (H1), first-instar larvae at 12 °C (M1), and first-instar larvae at 4 °C (L1).

**Additional file 6: Figure S4**. The series-clusters for DEGs in the second-instar larvae stage. Each cluster of DEGs showed similar expression change in second-instar larvae at 20 °C (H2), second-instar larvae at 12 °C (M2), and second-instar larvae at 4 °C (L2).

**Additional file 7: Figure S5**. The series-clusters for DEGs in the second-instar larvae stage. Each cluster of DEGs showed similar expression change in third-instar larvae at 20 °C (H3), third-instar larvae at 12 °C (M3), and third-instar larvae at 4 °C (L3).

**Additional file 8: Figure S6**. Functional annotation of assembled sequences of DEGs of first-instar larvae at 20 °C (H1) vs first-instar larvae at 4 °C (L1) based on gene ontology (GO) categorization. Unigenes were annotated in three categories: biological process, cellular components, and molecular functions.

**Additional file 9: Table S3**. GO functional enrichment analysis related with temperature of the DEGs of H1 vs L1, H1 vs M1 and M1 vs L1.

**Additional file 10: Figure S7.** KEGG significant enrichment analysis for DEGs between first-instar larvae at 20 °C (H1) and first-instar larvae at 4 °C (L1) of *A. grahami*.

**Additional file 11: Figure S8**. Functional annotation of assembled sequences of DEGs of second-instar larvae at 20 °C (H2) vs second-instar larvae at 4 °C (L2) based on gene ontology (GO) categorization. Unigenes were annotated in three categories: biological process, cellular components, and molecular functions.

**Additional file 12: Table S4**. GO functional enrichment analysis related with temperature of the DEGs of H2 vs L2, H2 vs M2 and M2 vs L2.

**Additional file 13: Figure S9**. KEGG significant enrichment analysis for DEGs between second-instar larvae at 20 °C (H2) and second-instar larvae at 4 °C (L2) of *A. grahami*.

**Additional file 14: Figure S10**. Functional annotation of assembled sequences of DEGs of third-instar larvae at 20 °C (H3) vs third-instar larvae at 4 °C (L3) based on gene ontology (GO) categorization. Unigenes were annotated in three categories: biological process, cellular components, and molecular functions.

**Additional file 15: Table S5**. GO functional enrichment analysis related with temperature of the DEGs of H3 vs L3, H3 vs M3 and M3 vs L3.

**Additional file 16: Figure S11**. KEGG significant enrichment analysis for DEGs between third-instar larvae at 20 °C (H3) and third-instar larvae at 4 °C (L3) of *A. grahami*.

**Additional file 17: Figure S12**. The gene co-expression net-work of DEGs in the middle temperatures (12 °C) was analyzed. (a) the gene co-expression net-work in egg stage at 12 °C (M0), (b) the gene co-expression net-work in first-instar larvae stage at 12 °C (M1), (c) the gene co-expression net-work in second-instar larvae stage at 12 °C (M2), (d) the gene co-expression net-work in third-instar larvae stage at 12 °C (M3).

**Additional file 18: Figure S13**. The gene co-expression net-work of DEGs in the relatively high temperatures (20 °C) was analyzed. (a) the gene co-expression net-work in egg stage at 20 °C (H0), (b) the gene co-expression net-work in first-instar larvae stage at 20 °C (H1), (c) the gene co-expression net-work in second-instar larvae stage at 20 °C (H2), (d) the gene co-expression net-work in third-instar larvae stage at 20 °C (H3).

**Additional file 19: Table S6**. Primers used for qPCR validation.

## Data Availability

The raw reads has been submitted to SRA at NCBI under accession number PRJNA565270 (https://www.ncbi.nlm.nih.gov/bioproject/PRJNA565270/). *Aldrichina grahami* genome sequence data: NCBI: PRJNA513084.

## Abbreviations

CHI10: Chitinase 10
CPs: Cuticular proteins
CU01: Cuticle protein 1
CUP: Pupal cuticle protein
CUP7: Pupal cuticle protein 7
CUP9: Pupal cuticle protein 7
DEGs: Differentially expressed genes
FPKM: Fragment per kilobase of exon model per million mapped reads
GO: Gene ontology
H0: Egg stage of *Aldrichina grahami* at 20 °C
H1: First-instar larvae stage of *Aldrichina grahami* at 20 °C
H2: Second-instar larvae stage of *Aldrichina grahami* at 20 °C
H3: Third-instar larvae stage of *Aldrichina grahami* at 20 °C
HSP: Heat shock proteins
HSP68: Heat shock protein 68
KEGG: Kyoto encyclopedia of genes and genomes
L0: Egg stage of *Aldrichina grahami* at 4 °C
L1: First-instar larvae stage of *Aldrichina grahami* at 4 °C
L2: Second-instar larvae stage of *Aldrichina grahami* at 4 °C
L3: Third-instar larvae stage of *Aldrichina grahami* at 4 °C
LCP: Larval cuticle proteins
LCP2: Larval cuticle protein 2
LCP5: Larval cuticle protein 5
LCP8: Larval cuticle protein 8
LT_50_: Lethal time of 50%
M0: Egg stage of *Aldrichina grahami* at 12 °C
M1: First-instar larvae stage of *Aldrichina grahami* at 12 °C
M2: Second-instar larvae stage of *Aldrichina grahami* at 12 °C
M3: Third-instar larvae stage of *Aldrichina grahami* at 12 °C
PMI_min_: Minimum postmortem interval
RT-qPCR: Quantitative real-time PCR
WAT: Fatty acyl-CoA reductase wat
