# Supplementary material for: Dynamic transcriptome profiling exploring cold tolerance in forensically important blow fly, Aldrichina grahami (Diptera: Calliphoridae)
Source: BMC Genomics. 2020 Jan 29;21:92. doi: 10.1186/s12864-020-6509-0 (PMC6988367; doi:10.1186/s12864-020-6509-0)
Supplement: Supplementary file 1 — Additional file 1: Table S1. Specimen collection of Aldrichina grahami. [file 12864_2020_6509_MOESM1_ESM.docx]

**Table S1.** Specimen collection of *Aldrichina grahami*

|  | Egg stage | First-instar  larvae stage | Second-instar larvae stage | Third-instar larvae stage |
| --- | --- | --- | --- | --- |
| 4℃ | L0A, L0B, L0C | L1A, L1B, L1C | L2A, L2B, L2C | L3A, L3B, L3C |
| 12℃ | M0A, M0B, M0C | M1A, M1B, M1C | M2A, M2B, M2C | M3A, M3B, M3C |
| 20℃ | H0A, H0B, H0C | H1A, H1B, H1C | H2A, H2B, H2C | H3A, H3B, H3C |
