# Supplementary figures and images for: Dynamic transcriptome profiling exploring cold tolerance in forensically important blow fly, Aldrichina grahami (Diptera: Calliphoridae)
Source: BMC Genomics. 2020 Jan 29;21:92. doi: 10.1186/s12864-020-6509-0 (PMC6988367; doi:10.1186/s12864-020-6509-0)

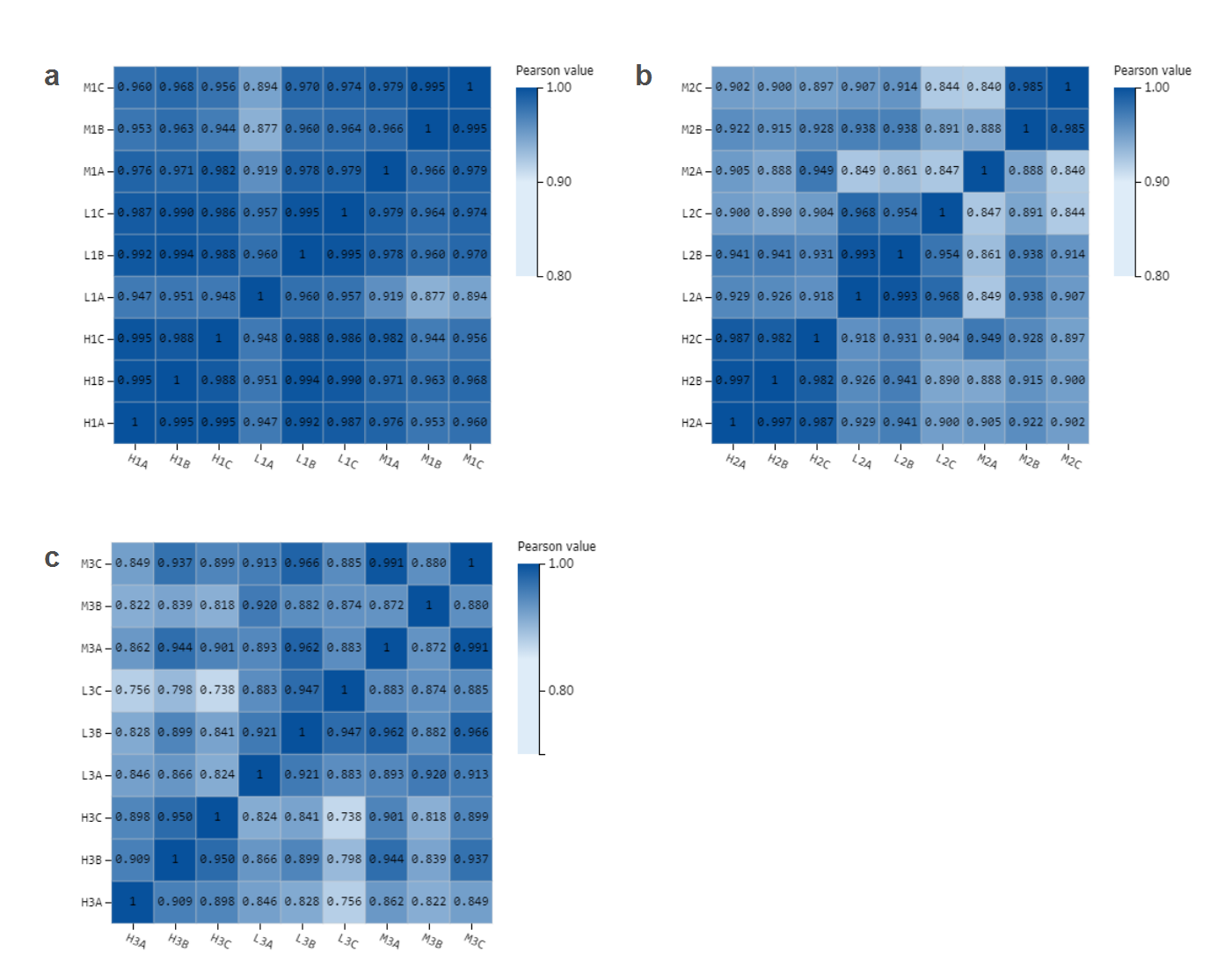

Supplement: Supplementary file 2 — Additional file 2: Figure S1. The Pearson correlation coefficient between three biological replicates. (a) the Pearson correlation coefficient between three biological replicates of first-instar larvae at 20 °C (H1), first-instar larvae at 12 °C (M1), and first-instar larvae at 4 °C (L1), (b) the Pearson correlation coefficient between three biological replicates of second-instar larvae at 20 °C (H2), second-instar larvae at 12 °C (M2), and second-instar larvae at 4 °C (L2), (c) the Pearson correlation coefficient between three biological replicates of third-instar larvae at 20 °C (H3), third-instar larvae at 12 °C (M3), and third-instar larvae at 4 °C (L3). [file 12864_2020_6509_MOESM2_ESM.png]

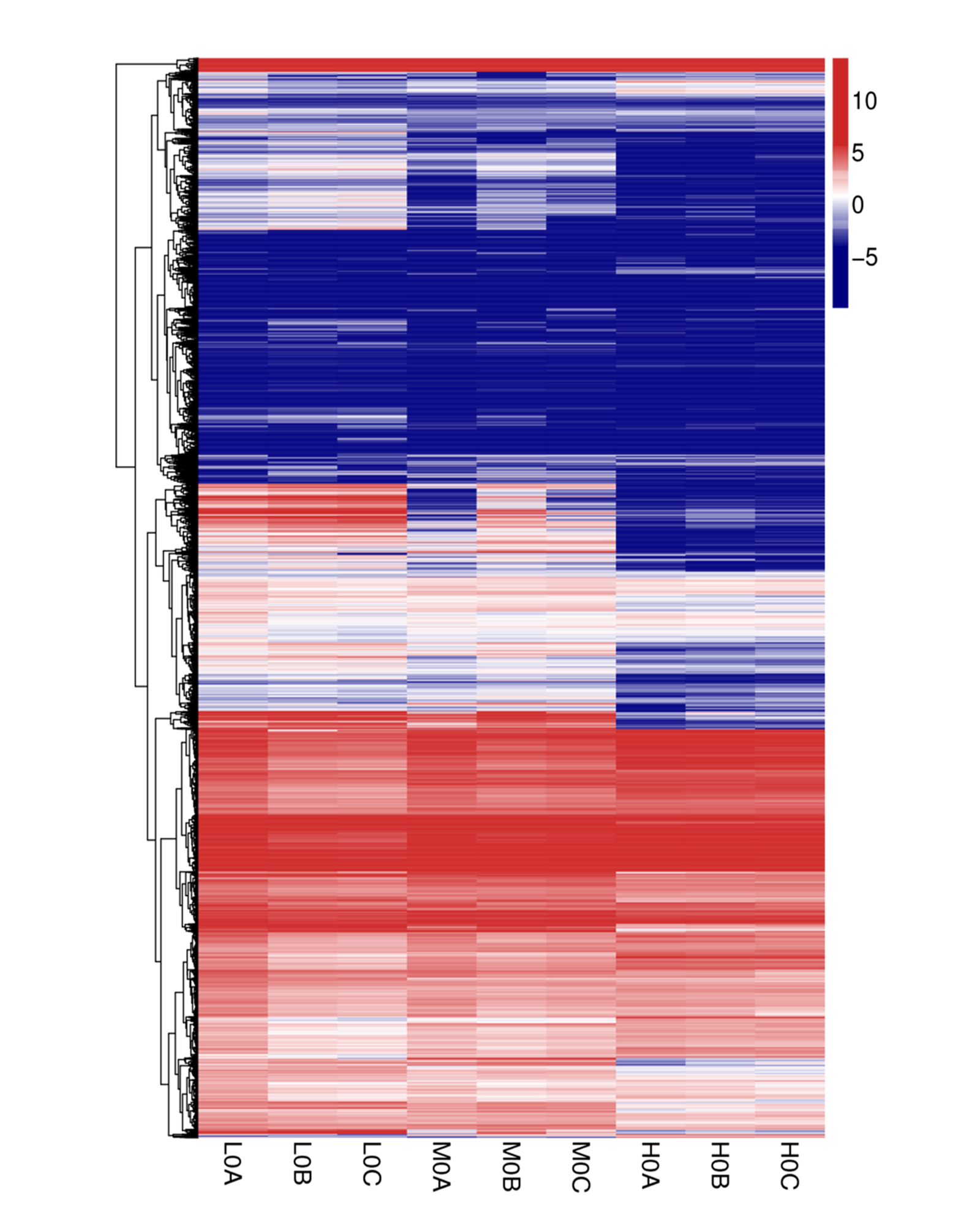

Supplement: Supplementary file 3 — Additional file 3: Figure S2. The expression patterns of all the DEGs under egg stage at 20 °C (H0), egg stage at 12 °C (M0), and egg stage at 4 °C (L0). [file 12864_2020_6509_MOESM3_ESM.png]

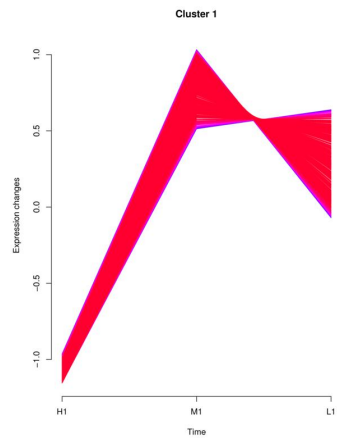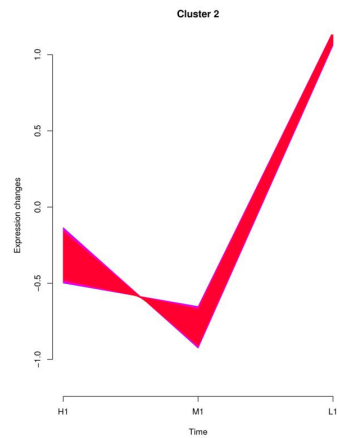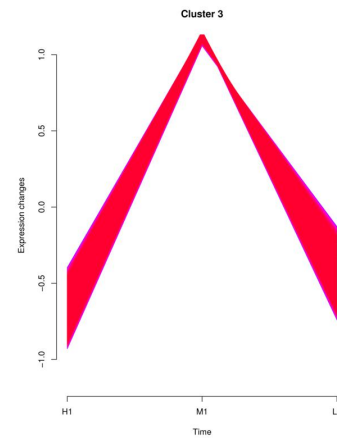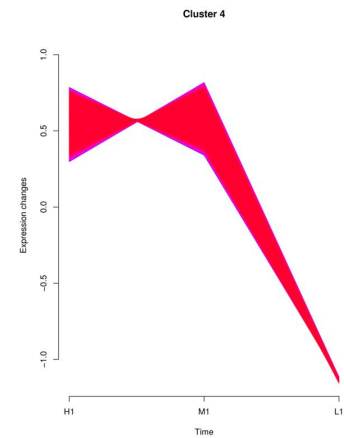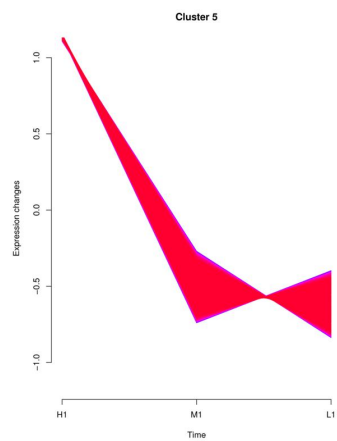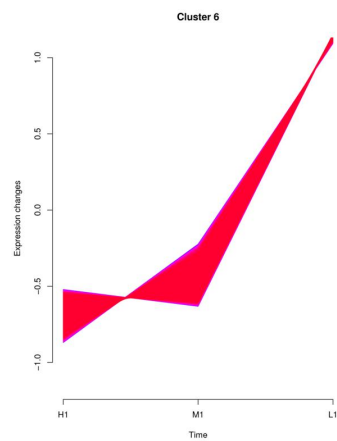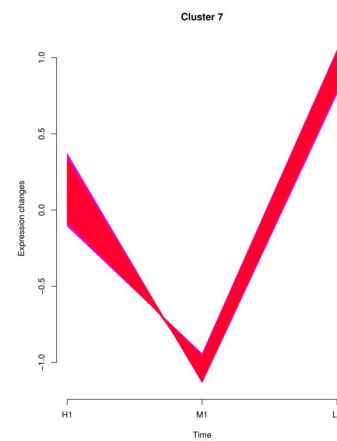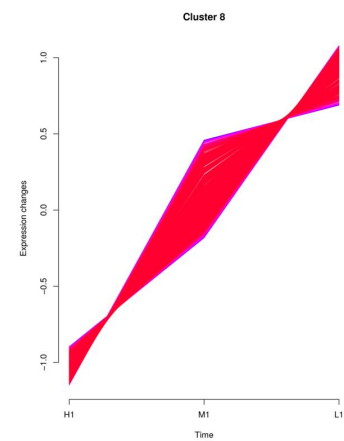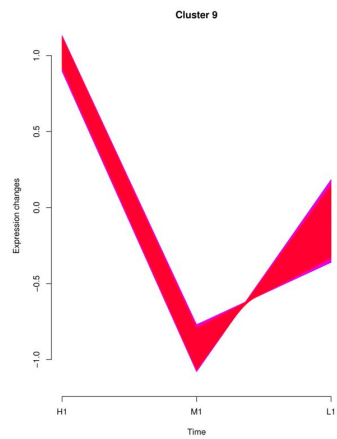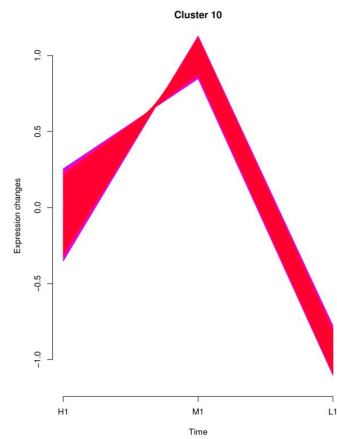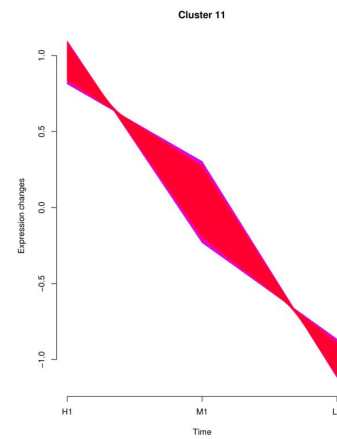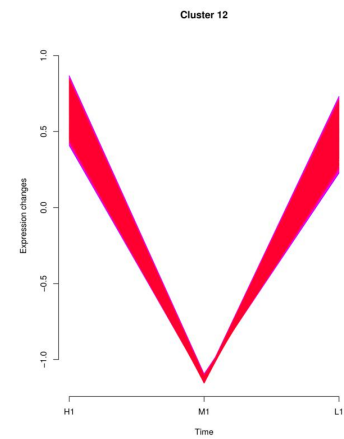

Supplement: Supplementary file 5 — Additional file 5: Figure S3. The series-clusters for DEGs in the first-instar larvae stage. Each cluster of DEGs showed similar expression change in first-instar larvae at 20 °C (H1), first-instar larvae at 12 °C (M1), and first-instar larvae at 4 °C (L1). [file 12864_2020_6509_MOESM5_ESM.pdf]

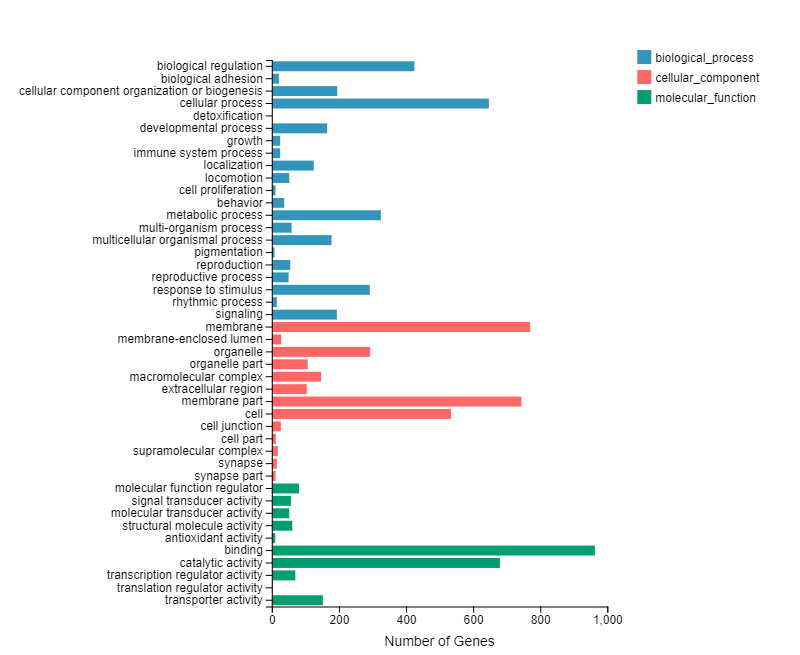

Supplement: Supplementary file 8 — Additional file 8: Figure S6. Functional annotation of assembled sequences of DEGs of first-instar larvae at 20 °C (H1) vs first-instar larvae at 4 °C (L1) based on gene ontology (GO) categorization. Unigenes were annotated in three categories: biological process, cellular components, and molecular functions. [file 12864_2020_6509_MOESM8_ESM.jpg]

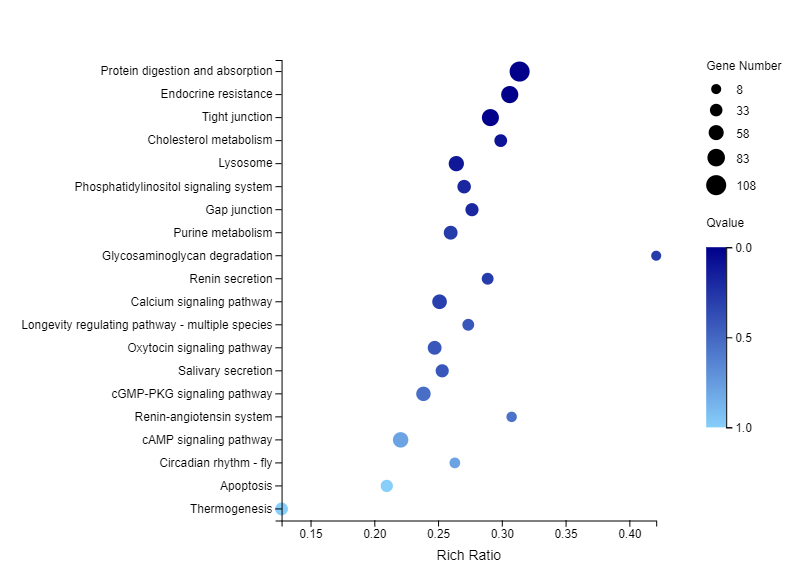

Supplement: Supplementary file 10 — Additional file 10: Figure S7. KEGG significant enrichment analysis for DEGs between first-instar larvae at 20 °C (H1) and first-instar larvae at 4 °C (L1) of A. grahami. [file 12864_2020_6509_MOESM10_ESM.jpg]

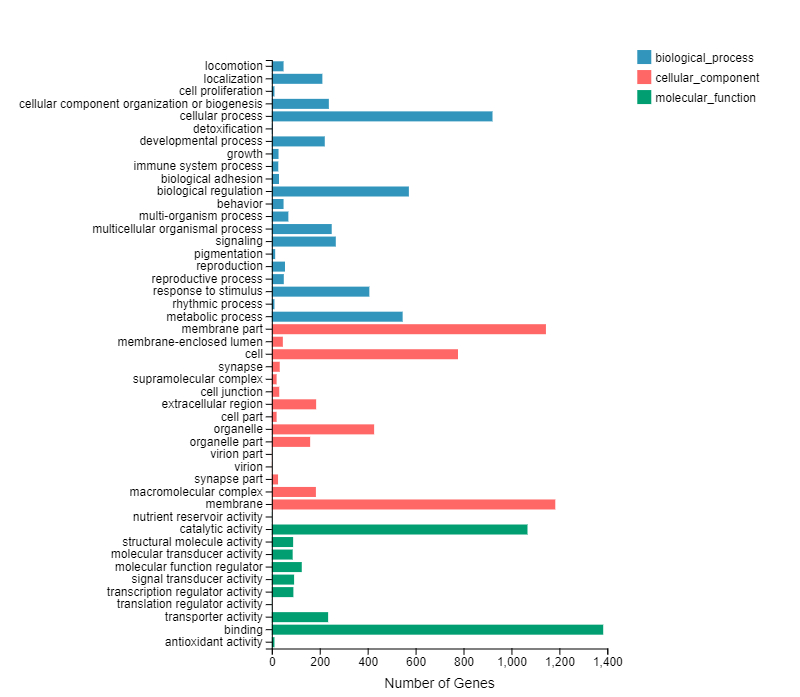

Supplement: Supplementary file 11 — Additional file 11: Figure S8. Functional annotation of assembled sequences of DEGs of second-instar larvae at 20 °C (H2) vs second-instar larvae at 4 °C (L2) based on gene ontology (GO) categorization. Unigenes were annotated in three categories: biological process, cellular components, and molecular functions. [file 12864_2020_6509_MOESM11_ESM.jpg]

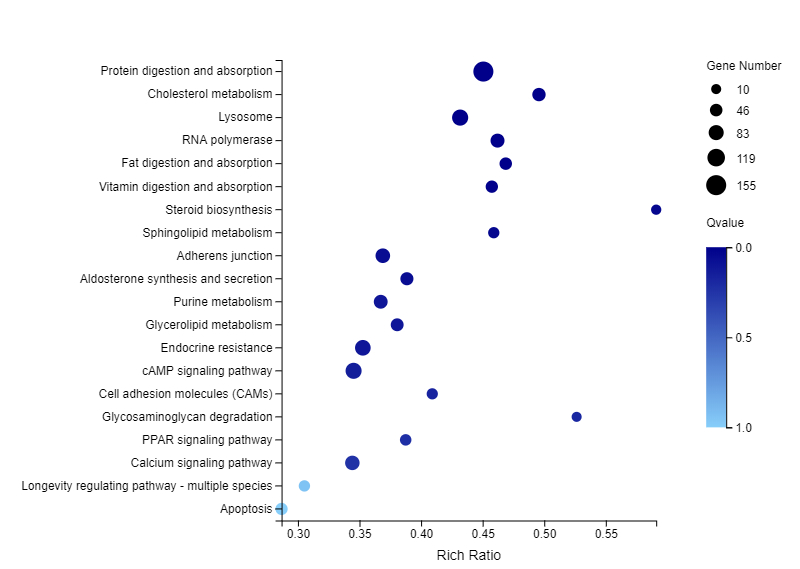

Supplement: Supplementary file 13 — Additional file 13: Figure S9. KEGG significant enrichment analysis for DEGs between second-instar larvae at 20 °C (H2) and second-instar larvae at 4 °C (L2) of A. grahami. [file 12864_2020_6509_MOESM13_ESM.jpg]

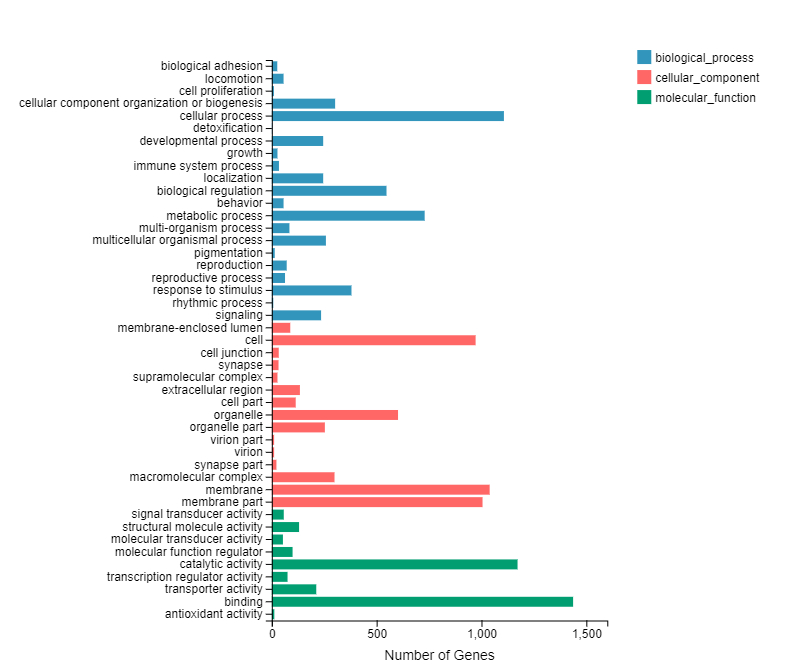

Supplement: Supplementary file 14 — Additional file 14: Figure S10. Functional annotation of assembled sequences of DEGs of third-instar larvae at 20 °C (H3) vs third-instar larvae at 4 °C (L3) based on gene ontology (GO) categorization. Unigenes were annotated in three categories: biological process, cellular components, and molecular functions. [file 12864_2020_6509_MOESM14_ESM.jpg]

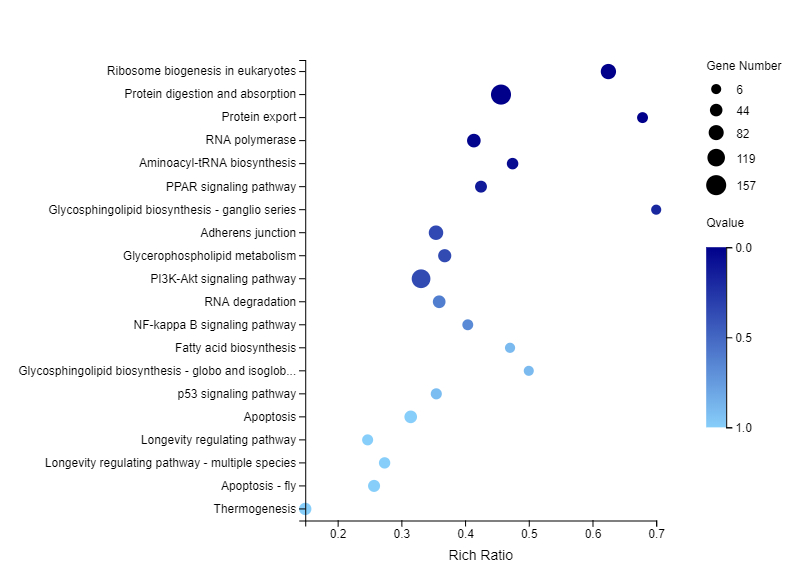

Supplement: Supplementary file 16 — Additional file 16: Figure S11. KEGG significant enrichment analysis for DEGs between third-instar larvae at 20 °C (H3) and third-instar larvae at 4 °C (L3) of A. grahami. [file 12864_2020_6509_MOESM16_ESM.jpg]

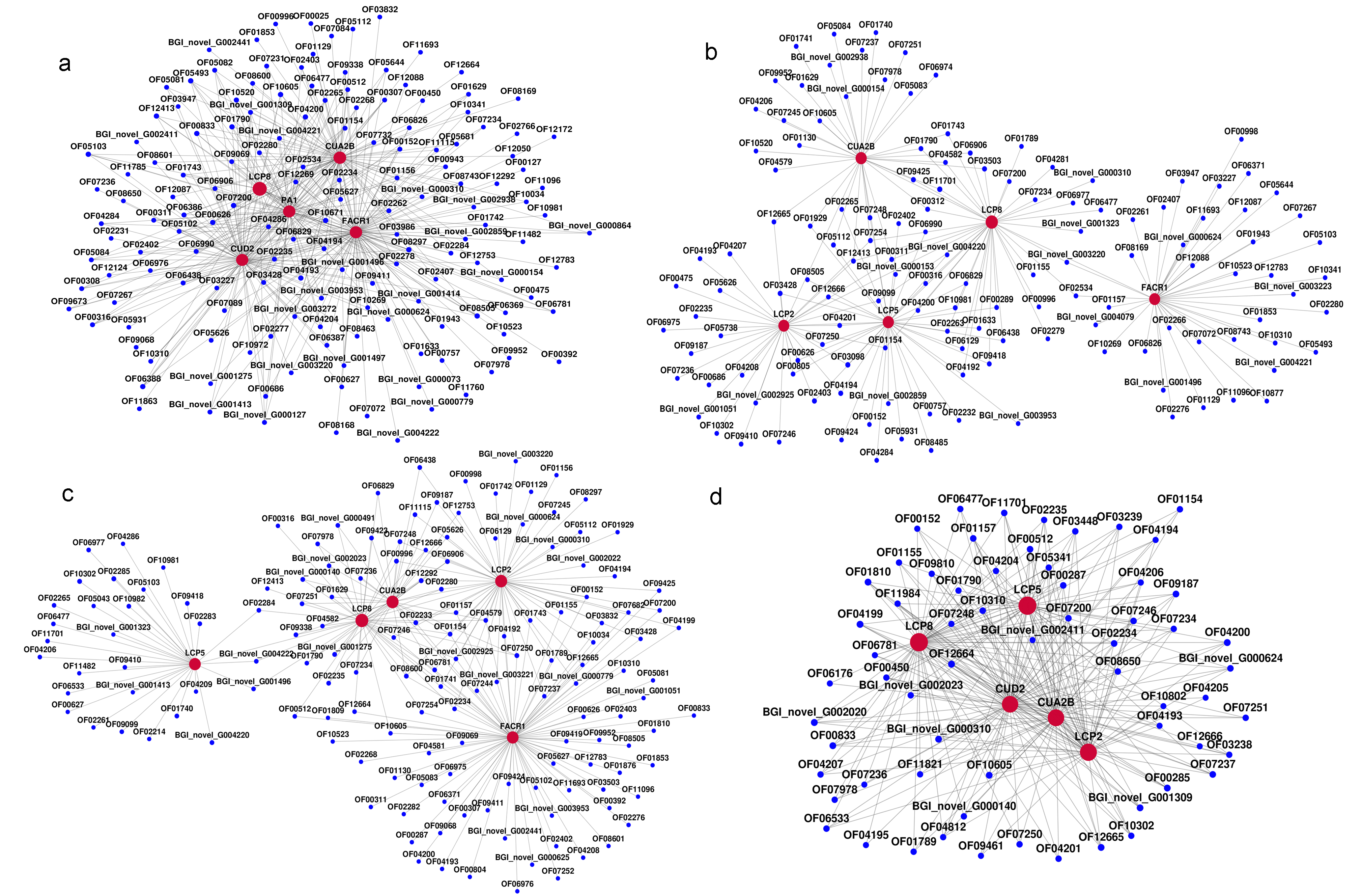

Supplement: Supplementary file 17 — Additional file 17: Figure S12. The gene co-expression net-work of DEGs in the middle temperatures (12 °C) was analyzed. (a) the gene co-expression net-work in egg stage at 12 °C (M0), (b) the gene co-expression net-work in first-instar larvae stage at 12 °C (M1), (c) the gene co-expression net-work in second-instar larvae stage at 12 °C (M2), (d) the gene co-expression net-work in third-instar larvae stage at 12 °C (M3). [file 12864_2020_6509_MOESM17_ESM.png]

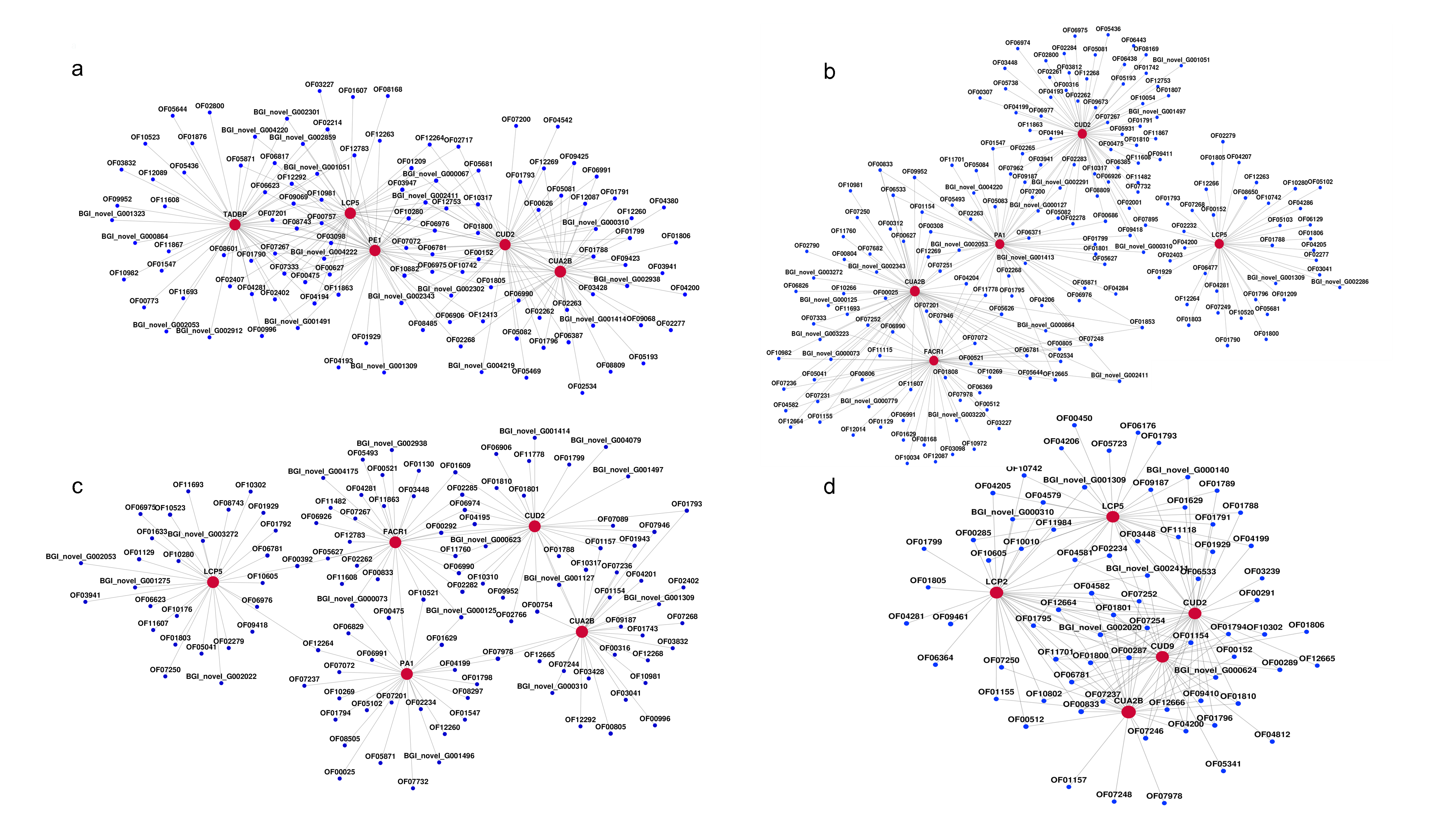

Supplement: Supplementary file 18 — Additional file 18: Figure S13. The gene co-expression net-work of DEGs in the relatively high temperatures (20 °C) was analyzed. (a) the gene co-expression net-work in egg stage at 20 °C (H0), (b) the gene co-expression net-work in first-instar larvae stage at 20 °C (H1), (c) the gene co-expression net-work in second-instar larvae stage at 20 °C (H2), (d) the gene co-expression net-work in third-instar larvae stage at 20 °C (H3). [file 12864_2020_6509_MOESM18_ESM.png]
