# Supplementary material for: Dynamic transcriptome profiling exploring cold tolerance in forensically important blow fly, Aldrichina grahami (Diptera: Calliphoridae)
Source: BMC Genomics. 2020 Jan 29;21:92. doi: 10.1186/s12864-020-6509-0 (PMC6988367; doi:10.1186/s12864-020-6509-0)
Supplement: Supplementary file 7 — Additional file 7: Figure S5. The series-clusters for DEGs in the second-instar larvae stage. Each cluster of DEGs showed similar expression change in third-instar larvae at 20 °C (H3), third-instar larvae at 12 °C (M3), and third-instar larvae at 4 °C (L3). [file 12864_2020_6509_MOESM7_ESM.pdf]

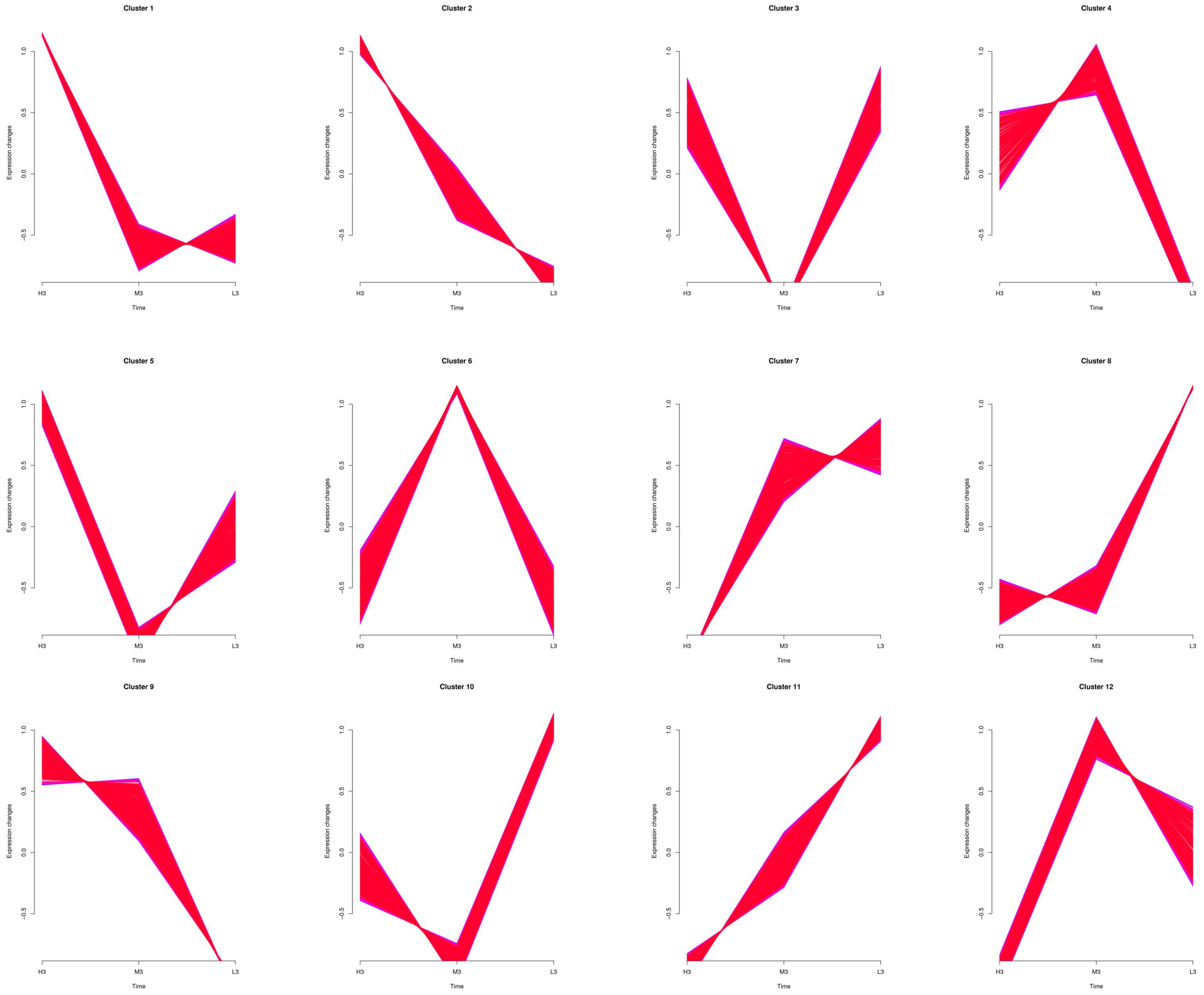

Cluster 1
Cluster 2
Cluster 3
Cluster 4
Cluster 5
Cluster 6
Cluster 7
Cluster 8
Cluster 9
Cluster 10
Cluster 11
Cluster 12
Expression changes
-0.5
0.0
0.5
1.0
H3
M3
L3
Time
