# Supplementary material for: Dynamic transcriptome profiling exploring cold tolerance in forensically important blow fly, Aldrichina grahami (Diptera: Calliphoridae)
Source: BMC Genomics. 2020 Jan 29;21:92. doi: 10.1186/s12864-020-6509-0 (PMC6988367; doi:10.1186/s12864-020-6509-0)
Supplement: Supplementary file 9 — Additional file 9: Table S3. GO functional enrichment analysis related with temperature of the DEGs of H1 vs L1, H1 vs M1 and M1 vs L1. [file 12864_2020_6509_MOESM9_ESM.docx]

**Table S3.** GO functional enrichment analysis related with temperature of the DEGs of H1vsL1, H1vsM1 and M1vsL1

| Tissue comparison | GO Term | Rich Ratio | P-value |
| --- | --- | --- | --- |
| H1vsL1 | response to stimulus | 0.21 | 3.23E-08 |
|  | regulation of Ras protein signal transduction | 0.33 | 5.00E-07 |
|  | circadian rhythm | 0.52 | 7.65E-06 |
|  | response to nutrient | 0.60 | 0.0001 |
|  | positive regulation of cytoskeleton organization | 0.41 | 0.0001 |
|  | regulation of glucose metabolic process | 0.32 | 0.0002 |
|  | structural constituent of cuticle | 0.28 | 0.0005 |
|  | chitin metabolic process | 0.23 | 0.0171 |
|  | glucosamine-containing  compound metabolic process | 0.22 | 0.0207 |
|  | amino sugar metabolic process | 0.22 | 0.0228 |
|  | chitin-based cuticle sclerotization | 0.33 | 0.0372 |
| H1vsM1 | structural constituent of cuticle | 0.51 | 1.93E-34 |
|  | chitin metabolic process | 0.26 | 1.92E-08 |
|  | glucosamine-containing  compound metabolic process | 0.26 | 2.87E-08 |
|  | amino sugar metabolic process | 0.26 | 3.50E-08 |
|  | aminoglycan metabolic process | 0.23 | 2.82E-07 |
|  | cell communication | 0.15 | 1.17E-06 |
|  | chitin binding | 0.25 | 2.86E-06 |
|  | response to stimulus | 0.13 | 0.0003 |
|  | G-protein coupled receptor signaling pathway | 0.24 | 0.0004 |
|  | NAD+ kinase activity | 0.32 | 0.0020 |
| M1vsL1 | cell communication | 0.29 | 3.38E-15 |
|  | response to stimulus | 0.27 | 1.44E-13 |
|  | structural constituent of cuticle | 0.40 | 7.68E-08 |
|  | chitin metabolic process | 0.35 | 7.10E-06 |
|  | aminoglycan metabolic process | 0.34 | 7.64E-06 |
|  | glucosamine-containing  compound metabolic process | 0.35 | 1.11E-05 |
|  | amino sugar metabolic process | 0.34 | 1.37E-05 |
|  | G-protein coupled receptor activity | 0.37 | 3.59E-05 |
|  | regulation of glucose metabolic process | 0.36 | 0.0004 |
|  | chitin binding | 0.32 | 0.0005 |
|  | growth | 0.31 | 0.0005 |
|  | circadian rhythm | 0.38 | 0.0017 |
