# Supplementary material for: Dynamic transcriptome profiling exploring cold tolerance in forensically important blow fly, Aldrichina grahami (Diptera: Calliphoridae)
Source: BMC Genomics. 2020 Jan 29;21:92. doi: 10.1186/s12864-020-6509-0 (PMC6988367; doi:10.1186/s12864-020-6509-0)
Supplement: Supplementary file 12 — Additional file 12: Table S4. GO functional enrichment analysis related with temperature of the DEGs of H2 vs L2, H2 vs M2 and M2 vs L2. [file 12864_2020_6509_MOESM12_ESM.docx]

**Table S4.** GO functional enrichment analysis related with temperature of the DEGs of H2vsL2, H2vsM2 and M2vsL2

| Tissue comparison | GO Term | Rich Ratio | P-value |
| --- | --- | --- | --- |
| H2vsL2 | aminoglycan metabolic process | 0.53 | 2.74E-17 |
|  | chitin metabolic process | 0.53 | 6.51E-15 |
|  | glucosamine-containing  compound metabolic process | 0.52 | 1.74E-14 |
|  | amino sugar metabolic process | 0.52 | 2.82E-14 |
|  | chitin binding | 0.51 | 1.78E-11 |
|  | structural constituent of cuticle | 0.46 | 1.00E-07 |
|  | response to stimulus | 0.29 | 2.16E-07 |
|  | site of DNA damage | 0.72 | 1.03E-06 |
|  | lipid metabolic process | 0.35 | 1.18E-06 |
|  | regulation of Ras protein signal transduction | 0.41 | 3.16E-06 |
|  | cell communication | 029 | 6.08E-06 |
|  | chitin-based cuticle sclerotization | 0.57 | 0.0009 |
| H2vsM2 | structural constituent of cuticle | 0.60 | 1.07E-33 |
|  | lipid metabolic process | 0.26 | 2.29E-08 |
|  | chitin metabolic process | 0.29 | 4.35E-06 |
|  | glucosamine-containing  compound metabolic process | 0.29 | 6.37E-06 |
|  | G-protein coupled receptor signaling pathway | 0.35 | 7.56E-06 |
|  | amino sugar metabolic process | 0.28 | 7.69E-06 |
|  | chitin binding | 0.26 | 0.0024 |
|  | response to stimulus | 0.17 | 0.0053 |
|  | cell communication | 0.17 | 0.0055 |
|  | fatty acid metabolic process | 0.29 | 0.0056 |
| M2vsL2 | aminoglycan metabolic process | 0.47 | 3.97E-14 |
|  | chitin metabolic process | 0.46 | 7.38E-12 |
|  | glucosamine-containing  compound metabolic process | 0.46 | 1.60E-11 |
|  | amino sugar metabolic process | 0.45 | 2.34E-11 |
|  | structural constituent of cuticle | 0.47 | 1.38E-10 |
|  | response to stimulus | 0.27 | 3.90E-10 |
|  | chitin binding | 0.45 | 4.54E-09 |
|  | cell communication | 0.28 | 6.88E-09 |
|  | G-protein coupled receptor activity | 0.5 | 7.06E-08 |
|  | circadian rhythm | 0.59 | 1.14E-05 |
|  | lipid metabolic process | 0.29 | 0.0006 |
