# Supplementary material for: Dynamic transcriptome profiling exploring cold tolerance in forensically important blow fly, Aldrichina grahami (Diptera: Calliphoridae)
Source: BMC Genomics. 2020 Jan 29;21:92. doi: 10.1186/s12864-020-6509-0 (PMC6988367; doi:10.1186/s12864-020-6509-0)
Supplement: Supplementary file 15 — Additional file 15: Table S5. GO functional enrichment analysis related with temperature of the DEGs of H3 vs L3, H3 vs M3 and M3 vs L3. [file 12864_2020_6509_MOESM15_ESM.docx]

**Table S5.** GO functional enrichment analysis related with temperature of the DEGs of H3vsL3, H3vsM3 and M3vsL3

| Tissue comparison | GO Term | Rich Ratio | P-value |
| --- | --- | --- | --- |
| H3vsL3 | rRNA metabolic process | 0.73 | 8.86E-08 |
|  | structural constituent of cuticle | 0.44 | 1.58E-06 |
|  | tRNA metabolic process | 0.48 | 4.32E-06 |
|  | response to starvation | 0.54 | 1.98E-05 |
|  | glycogen metabolic process | 0.65 | 0.0003 |
|  | regulation of Ras protein signal transduction | 0.39 | 0.0007 |
|  | cuticle pigmentation | 0.59 | 0.0011 |
|  | glycogen biosynthetic process | 0.73 | 0.0017 |
|  | chitin-based cuticle sclerotization | 0.57 | 0.0026 |
|  | adult chitin-containing cuticle pigmentation | 0.57 | 0.0026 |
|  | glycosaminoglycan binding | 0.54 | 0.0030 |
| H3vsM3 | structural constituent of cuticle | 0.46 | 3.08E-18 |
|  | glucosamine-containing  compound metabolic process | 0.27 | 5.59E-05 |
|  | amino sugar metabolic process | 0.27 | 6.59E-05 |
|  | chitin metabolic process | 0.27 | 9.10E-05 |
|  | aminoglycan metabolic process | 0.26 | 0.0001 |
|  | response to stimulus | 0.16 | 0.0043 |
|  | chitin binding | 0.25 | 0.0047 |
|  | regulation of lipid metabolic process | 0.39 | 0.011 |
|  | glycogen metabolic process | 0.40 | 0.053 |
|  | energy reserve metabolic process | 0.40 | 0.053 |
| M3vsL3 | rRNA processing | 0.72 | 4.70E-12 |
|  | cellular response to starvation | 0.50 | 3.51E-07 |
|  | response to nutrient levels | 0.40 | 4.94E-07 |
|  | cell communication | 0.19 | 2.31E-05 |
|  | response to starvation | 0.37 | 9.50E-05 |
|  | glycosyl compound biosynthetic process | 0.5 | 0.0005 |
|  | response to nutrient | 0.53 | 0.0006 |
|  | cell cycle DNA replication | 0.50 | 0.0010 |
|  | response to stimulus | 0.17 | 0.0015 |
|  | cAMP binding | 0.50 | 0.0027 |
