# Supplementary material for: Dynamic transcriptome profiling exploring cold tolerance in forensically important blow fly, Aldrichina grahami (Diptera: Calliphoridae)
Source: BMC Genomics. 2020 Jan 29;21:92. doi: 10.1186/s12864-020-6509-0 (PMC6988367; doi:10.1186/s12864-020-6509-0)
Supplement: Supplementary file 19 — Additional file 19: Table S6. Primers used for qPCR validation. [file 12864_2020_6509_MOESM19_ESM.docx]

| **Table S6.** Primers used for qPCR validation |
| --- |

| Gene | Primer sequences (5'-3') | Product size (bp) |
| --- | --- | --- |
| OF01796 | F:GTTACGAAACCAGCGATGGC  R:CGGCAACATAGTTGACGGTG | 144 |
| OF01805 | F:TTCGTTGCTCTCTTCGCCAT  R:TGTTCGGAGCCAACATTGTG | 152 |
| BGI_novel_G000249 | F: CCGTCGGTCGTATTCATCGT R:TCAAGAACTTCAGCGGCCAA | 115 |
| OF05523 | F:GGCCTTATCTGCCGCTACAA  R:ATGGTGGTTGCGAACATCCT | 153 |
| OF12392 | F:CAACAAGTGGTGCAGCAGTC  R:CCATATTTTGAGCCAGCCGC | 149 |
| OF03984 | F:CCCGAACACAAACACACCAA  R:AACCGTGACGTACATTTGCC | 148 |
| OF02256 | F:CATGCCAGCCAACAAACCTC R:TCGCTGGAGTAATCGGCATC | 147 |
| OF05966 | F:GGGTCATGGAGAAATGCGAA R:AGTCTGGTCATGATGGGCTG | 124 |
| OF01804 | F:GGCCAAACCAGCTGAAGTTG  R:CGTCTTCATGGCGGGAAGTA | 165 |
